# Author Correction: Development and validation of an age-sex-ethnicity-specific metabolic syndrome score in the Chinese adults

**DOI:** 10.1038/s41467-024-46992-4

**Published:** 2024-03-22

**Authors:** Shujuan Yang, Bin Yu, Wanqi Yu, Shaoqing Dai, Chuanteng Feng, Ying Shao, Xing Zhao, Xiaoqing Li, Tianjing He, Peng Jia

**Affiliations:** 1 West China School of Public Health and West China Fourth Hospital, Sichuan University, Chengdu, China; 2 International Institute of Spatial Lifecourse Health (ISLE), Wuhan University, Wuhan, China; 3 Institute for Disaster Management and Reconstruction, Sichuan University, Chengdu, China; 4 Faculty of Geo-information Science and Earth Observation, University of Twente, Enschede, the Netherlands; 5 Yunnan Center for Disease Control and Prevention, Kunming, China; 6 Fujian Provincial Center for Disease Control and Prevention, Fuzhou, China; 7 Hubei Provincial Center for Disease Control and Prevention, Wuhan, China; 8 School of Resource and Environmental Sciences, Wuhan University, Wuhan, China; 9 Hubei Luojia Laboratory, Wuhan, China; 10 School of Public Health, Wuhan University, Wuhan, China

**Keywords:** Metabolic syndrome, Risk factors, Metabolic syndrome

Correction to: *Nature Communications* 10.1038/s41467-023-42423-y, published 01 November 2023

The original version of this article incorrectly referenced Tables S3, S5, S6, S10, S14 in the main Article, which were previously cited as Tables S17, S4, S5, S9, S13, respectively.

The original version of this article contained a spelling mistake in the affiliations on the first page, “Hubei Loujia Laboratory” should read “Hubei Luojia Laboratory”.

These errors have been corrected in both the PDF and HTML versions of the Article.

