# Supplementary material for: Development and validation of an age-sex-ethnicity-specific metabolic syndrome score in the Chinese adults
Source: Nat Commun. 2023 Nov 1;14:6988. doi: 10.1038/s41467-023-42423-y (PMC10620391; doi:10.1038/s41467-023-42423-y)
Supplement: Supplementary file 1 — Supplementary Information [file 41467_2023_42423_MOESM1_ESM.pdf]

Development and validation of an age-sex-ethnicity-specific metabolic syndrome score in the Chinese adults

Table S1. Characteristics of the participants in the China Multi-Ethnic Cohort (CMEC) baseline survey 2

Table S2. Association between the metabolic syndrome (MetS) score and cardiovascular disease (CVD)-related risk factors and risk markers in the China Multi-Ethnic Cohort (CMEC) baseline survey..... 4

Table S3. Characteristics of the participants in the China Multi-Ethnic Cohort (CMEC) follow-up survey.. 5

Table S4. Associations between the metabolic syndrome (MetS) score and the cardiovascular disease (CVD)-related risk factors and risk markers with the C-index in the China Multi-Ethnic Cohort (CMEC) follow-up survey 7

Table S5. Capacities of the traditionally defined metabolic syndrome (MetS) and the dichotomous age-sex-ethnicity-specific MetS score in detecting one or more cardiovascular disease (CVD)-related risk factors and risk markers in the China Multi-Ethnic Cohort (CMEC) dataset 8

Table S6. Characteristics of the participants in the Yunnan Behavior and Disease Surveillance (YBDS) survey 10

Table S7. Associations between the metabolic syndrome (MetS) score and the cardiovascular disease (CVD)-related risk factors and risk markers in the Yunnan Behavior and Disease Surveillance (YBDS) survey 12

Table S8. Associations between the metabolic syndrome (MetS) score in quartiles and the cardiovascular disease (CVD)-related risk factors and risk markers in the Yunnan Behavior and Disease Surveillance (YBDS) survey 13

Table S9. Capacities of the traditionally defined metabolic syndrome (MetS) and the dichotomous age-sex-ethnicity-specific MetS in detecting one or more cardiovascular disease (CVD)-related risk factors in the Yunnan Behavior and Disease Surveillance (YBDS) survey 15

Table S10. Characteristics of the participants in the Hubei Behavior and Disease Surveillance (HBDS) survey 17

Table S11. Associations between the metabolic syndrome (MetS) score and the cardiovascular disease (CVD)-related risk factors and risk markers in the Hubei Behavior and Disease Surveillance (HBDS) survey 19

Table S12. Associations between the metabolic syndrome (MetS) score in quartiles and the cardiovascular disease (CVD)-related risk factors and risk markers in the Hubei Behavior and Disease Surveillance (HBDS) survey 20

Table S13. Capacities of the traditionally defined metabolic syndrome (MetS) and the dichotomous age-sex-ethnicity-specific MetS in detecting one or more cardiovascular disease (CVD)-related risk factors in the Hubei Behavior and Disease Surveillance (HBDS) survey 21

Table S14. Characteristics of the participants in the Fujian Behavior and Disease Surveillance (FBDS) survey 22

Table S15. Associations between the metabolic syndrome (MetS) score and the cardiovascular disease (CVD)-related risk factors and risk markers in the Fujian Behavior and Disease Surveillance (FBDS) survey 24

Table S16. Associations between the metabolic syndrome (MetS) score in quartiles and the cardiovascular disease (CVD)-related risk factors and risk markers in the Fujian Behavior and Disease Surveillance (FBDS) survey 25

Table S17. Capacities of the traditionally defined metabolic syndrome (MetS) and the dichotomous age-sex-ethnicity-specific MetS in detecting one or more cardiovascular disease (CVD)-related risk factors in the Fujian Behavior and Disease Surveillance (FBDS) survey 26

Figure S1. A flowchart of participant enrolment. 27

Table S1. Characteristics of the participants in the China Multi-Ethnic Cohort (CMEC) baseline survey

Variables	Overall (N=77,639)	Han Male <60 yrs (N=14,651)	Han Male ≥60 yrs (N=4,712)	Han Female < 60 yrs (N=20,611)	Han Female ≥60 yrs (N=4,808)	Minority Male <60 yrs (N=8,186)	Minority Male ≥60 yrs (N=2,746)	Minority Female <60 yrs (N=17,833)	Minority Female ≥60 yrs (N=4,092)	*p*-value[a]
MetS components										
**Waist circumference** (cm)	81.8±10.1	84.5±9.3	82.7±9.3	77.6±8.7	81.5±9.6	86.7±10.5	83.3±10.6	81.8±10.4	82.3±11.0	0.013
**Triglycerides** (mmol/L)	1.6±1.5	2.0±1.9	1.4±1.1	1.4±1.2	1.7±1.1	2.0±2.1	1.5±1.2	1.4±1.2	1.6±1.0	<0.001
**HDL-C** (mmol/L)	1.5±0.4	1.3±0.4	1.5±0.4	1.5±0.4	1.6±0.4	1.4±0.4	1.5±0.4	1.5±0.4	1.6±0.4	<0.001
**Mean arterial pressure** (mmHg)	93.7±12.5	96.1±11.9	98.7±12.5	89.7±11.5	96.5±12.2	96.5±12.5	99.7±13.3	91.3±11.9	91.3±11.9	<0.001
**Fasting blood glucose** (mmol/L)	5.2±1.1	5.3±1.3	5.5±1.1	5.1±0.8	5.5±1.0	5.2±1.4	5.4±1.2	5.1±0.9	5.3±1.1	<0.001
CVD-related risk factors										
**Diabetes**										<0.001
No	72,694 (93.6)	13,641 (93.1)	4,203 (89.2)	19,910 (96.6)	4,351 (90.5)	7,490 (91.5)	2,411 (87.8)	17,030 (95.5)	3,658 (89.4)	
Yes	4,945 (6.4)	1,010 (6.9)	509 (10.8)	701 (3.4)	457 (9.5)	696 (8.5)	335 (12.2)	803 (4.5)	434 (10.6)	
**Hypertension**										<0.001
No	60,169 (77.5)	11,157 (76.2)	2,808 (59.6)	17,890 (86.8)	2,969 (61.8)	6,030 (73.7)	1,633 (59.5)	15,039 (84.3)	2,643 (64.6)	
Yes	17,470 (22.5)	3,494 (23.8)	1,904 (40.4)	2,721 (13.2)	1,839 (38.2)	2,156 (26.3)	1,113 (40.5)	2,794 (15.7)	1,449 (35.4)	
**Hyperlipidemia**										<0.001
No	56,410 (72.7)	9,152 (62.5)	3,599 (76.4)	16,679 (80.9)	3,354 (69.8)	4,894 (59.8)	1,942 (70.7)	13,966 (78.3)	2,824 (69.0)	
Yes	21,229 (27.3)	5,499 (37.5)	1,113 (23.6)	3,932 (19.1)	1,454 (30.2)	3,292 (40.2)	804 (29.3)	3,867 (21.7)	1,268 (31.0)	
CVD-related risk markers										
**HbA1c** (mmol/mol)	5.7±0.7	5.6±0.8	5.8±0.7	5.5±0.6	5.9±0.7	5.8±0.9	5.9±0.9	5.6±0.6	5.9±0.8	<0.001
**Total cholesterol** (mmol/L)	5.0±1.0	5.0±1.0	5.0±1.0	4.8±0.9	5.4±1.0	5.1±1.0	5.1±1.0	4.9±1.0	5.3±1.0	<0.001
**Body mass index** (kg/m^2)	24.0±3.4	24.5±3.3	23.6±3.1	23.5±3.2	24.0±3.5	24.6±3.6	23.1±3.4	24.2±3.6	23.3±3.8	<0.001
**Serum uric acid** (μmol/L)	314.8±87.0	373.7±83.1	351.5±80.1	274.9±61.8	291.7±68.5	380.2±87.3	372.0±90.8	275.9±67.4	290.3±75.3	<0.001
Covariates										
**Marital status**										<0.001
Married	69,482 (89.5)	13,475 (92.0)	4,268 (90.6)	18,834 (91.3)	3,666 (76.3)	7,531 (92.0)	2,410 (87.7)	16,378 (91.8)	2,920 (71.4)	
Unmarried/divorced/widowed	8,157 (10.5)	1,176 (8.0)	444 (9.4)	1,777 (8.7)	1,142 (23.7)	655 (8.0)	336 (12.3)	1,455 (8.2)	1,172 (28.6)	
**Educational level**										<0.001

High school or above	18,075 (23.3)	6,049 (41.3)	758 (16.1)	7,147 (34.7)	462 (9.6)	1,408 (17.2)	284 (10.3)	1,855 (10.4)	112 (2.7)	
Primary and junior high school	39,707 (51.1)	8,028 (54.8)	3,027 (64.2)	11,446 (55.5)	2,278 (47.4)	4,622 (56.5)	1,363 (49.7)	8,146 (45.7)	797 (19.5)	
Illiteracy	19,857 (25.6)	574 (3.9)	927 (19.7)	2,018 (9.8)	2,068 (43.0)	2,156 (26.3)	1,099 (40.0)	7,832 (43.9)	3,183 (77.8)	
**Annual family income** (*yuan*)										<0.001
<12,000	13,087 (16.9)	1,270 (8.7)	974 (20.7)	1,939 (9.4)	1,108 (23.0)	1,610 (19.7)	960 (35.0)	3,679 (20.6)	1,547 (37.8)	
12,000-19,999	14,301 (18.4)	1,617 (11.0)	740 (15.7)	3,048 (14.8)	850 (17.7)	1,924 (23.5)	599 (21.8)	4,617 (25.9)	906 (22.1)	
20,000-59,999	28,338 (36.5)	5,308 (36.2)	1,669 (35.4)	7,979 (38.7)	1,713 (35.6)	3,097 (37.8)	761 (27.7)	6,699 (37.6)	1,112 (27.2)	
60,000-99,999	11,265 (14.5)	2,883 (19.7)	806 (17.1)	3,769 (18.3)	720 (15.0)	897 (11.0)	238 (8.7)	1,649 (9.2)	303 (7.4)	
≥100,000	10,648 (13.7)	3,573 (24.4)	523 (11.1)	3,876 (18.8)	417 (8.7)	658 (8.0)	188 (6.8)	1,189 (6.7)	224 (5.5)	
**Residential location**										<0.001
Urban	39,599 (51.0)	11,269 (76.9)	3,236 (68.7)	15,812 (76.7)	3,432 (71.4)	1,395 (17.0)	507 (18.5)	3,270 (18.3)	678 (16.6)	
Rural	38,040 (49.0)	3,382 (23.1)	1,476 (31.3)	4,799 (23.3)	1,376 (28.6)	6,791 (83.0)	2,239 (81.5)	14,563 (81.7)	3,414 (83.4)	
**Alcohol drinking**										<0.001
No	43,490 (56.0)	3,717 (25.4)	1,670 (35.4)	13,297 (64.5)	3,528 (73.4)	3,327 (40.6)	1,321 (48.1)	13,293 (74.5)	3,337 (81.6)	
Yes	34,149 (44.0)	10,934 (74.6)	3,042 (64.6)	7,314 (35.5)	1,280 (26.6)	4,859 (59.4)	1,425 (51.9)	4,540 (25.5)	755 (18.4)	
**Smoking**										<0.001
No	58,539 (75.4)	5,607 (38.3)	1,502 (31.9)	20,170 (97.8)	4,760 (99.0)	3,610 (44.1)	1,175 (42.8)	17,668 (99.1)	4,047 (98.9)	
Yes	19,100 (24.6)	9,044 (61.7)	3,210 (68.1)	441 (2.2)	48 (1.0)	4,576 (55.9)	1,571 (57.2)	165 (0.9)	45 (1.1)	
**Diet** (g/week)[b]										
Cereal	4,030.7±2,130.8	4,714.1±2,191.2	4,678.1±2,217.6	3,637.1±1,900.0	3,948.3±1,941.2	4,302.4±2,262.3	3,994.0±2,081.7	3,791.5±2,140.3	3,441.2±1,973.0	<0.001
Fruits	838.5±804.1	826.6±769.3	711.1±749.0	1129.3±857.1	851.6±813.3	571.6±674.6	513.9±655.9	770.5±770.6	596.3±708.8	<0.001
Vegetables	2,163.7±1,445.1	2,210.9±1,471.0	2,616.4±1,671.0	2,237.4±1,438.3	2,429.2±1,564.2	2,020.5±1,411.6	1,965.3±1,336.9	2,004.0±1,350.7	1,906.1±1,278.0	<0.001
Meat	667.4±641.4	791.7±664.2	681.9±629.5	531.8±481.8	468.9±461.3	872.8±797.9	705.2±682.0	692.9±692.2	575.2±621.8	0.014
Soybean products	73.8±126.9	80.4±134.7	75.6±128.0	77.8±132.1	68.9±127.3	72.0±123.2	65.2±113.8	70.0±120.4	59.8±109.2	<0.001
Aquatic products	100.3±157.4	173.4±205.9	123.2±171.9	126.5±166.4	81.5±132.4	62.8±114.3	60.5±112.1	47.0±94.7	36.4±82.0	<0.001
**Physical activity** (METs/week)[c]	27.1±18.3	28.3±17.7	19.7±17.4	26.7±17.1	20.3±16.1	30.3±20.3	21.9±18.7	30.7±18.8	21.7±16.9	<0.001

CVD, cardiovascular disease; HbA1c, glycated haemoglobin; HDL-C, high-density lipoprotein cholesterol; METs, metabolic equivalent for the tasks; MetS, metabolic syndrome; SD, standard deviation.

[a]Significance of the differences in the values among the eight age-sex-ethnicity-specific subgroups, tested by the two-sided analysis of variance for continuous variables and the two-sided Chi-square test for categorical variable. [b]Diet was surveyed as how many grams were consumed on average per week during the preceding 12 months, measured by a mold of the standard serving size. [c]Physical activity was measured by the METs for occupational, traffic-related, chore, and leisure time activities on average per week during the preceding 12 months.

Table S2. Association between the metabolic syndrome (MetS) score and cardiovascular disease (CVD)-related risk factors and risk markers in the China Multi-Ethnic Cohort (CMEC) baseline survey

Groups	OR (95% CI)			*β* (95% CI)			
	**Hyperlipidemia**	**Diabetes**	**Hypertension**	**HbA1c**	**CHOL**	**BMI**	**SUA**
**Overall**[a]	1.30 (1.29, 1.32)**	1.05 (1.05, 1.05)**	1.09 (1.07, 1.11)**	0.17 (0.10, 0.17)**	0.24 (0.23, 0.24)**	1.71 (1.69, 1.73)**	26.21 (25.70, 26.72)**
**Han**[b]							
**Male**							
<60	1.34 (1.31, 1.38)**	1.05 (1.05, 1.06)**	1.09 (1.05, 1.12)**	0.19 (0.17, 0.21)**	0.28 (0.26, 0.30)**	1.73 (1.66, 1.79)**	27.69 (25.97, 29.42)**
≥60	1.31 (1.25, 1.38)**	1.06 (1.05, 1.07)*	1.10 (1.04, 1.17)*	0.21 (0.19, 0.23)**	0.27 (0.25, 0.29)**	2.22 (2.16, 2.28)**	30.89 (29.09, 32.70)**
**Female**							
<60	1.30 (1.26, 1.34)**	1.03 (1.03, 1.04)**	1.06 (1.03, 1.09)**	0.20 (0.17, 0.24)**	0.17 (0.13, 0.21)**	1.96 (1.85, 2.07)**	25.93 (22.33, 29.54)**
≥60	1.21 (1.16, 1.27)**	1.05 (1.04, 1.06)**	1.01 (0.96, 1.06)	0.17 (0.14, 0.21)**	0.08 (0.04, 0.12)**	2.13 (2.03, 2.23)**	24.91 (21.53, 28.28)**
**Minority**[b]							
**Male**							
<60	1.33 (1.27, 1.40)**	1.07 (1.06, 1.07)**	1.12 (1.07, 1.18)**	0.16 (0.15, 0.17)**	0.25 (0.24, 0.27)**	1.87 (1.82, 1.92)**	25.61 (24.56, 26.66)**
≥60	1.14 (1.00, 1.29)	1.07 (1.06, 1.08)**	1.08 (0.95, 1.23)	0.14 (0.13, 0.15)**	0.30 (0.29, 0.31)**	1.34 (1.29, 1.39)**	21.33 (20.39, 22.27)**
**Female**							
<60	1.28 (1.24, 1.33)**	1.04 (1.04, 1.04)**	1.08 (1.05, 1.12)**	0.19 (0.16, 0.22)**	0.20 (0.16, 0.23)**	2.05 (1.93, 2.17)**	26.82 (24.20, 29.44)**
≥60	1.20 (1.12, 1.29)**	1.06 (1.05, 1.07)**	1.04 (0.96, 1.12)	0.14 (0.11, 0.16)**	0.05 (0.01, 0.08)*	1.67 (1.57, 1.78)**	22.10 (19.90, 24.30)**

BMI, body mass index; CHOL, cholesterol; CI, confidence interval; HbA1c, glycated hemoglobin; OR: odds ratio; SUA, serum uric acid.

[a]Adjusted for age, sex, ethnicity, marital status, educational level, annual family income, residential location, alcohol drinking, smoking, diet, and physical activity. [b]Adjusted for marital status, educational level, annual family income, residential location, alcohol drinking, smoking, diet, and physical activity.

OR and *β* are estimated by multiple logistic regression and linear regression, respectively, and all tests are two-sided.

*$p<0.05$, **$p<0.001$

Table S3. Characteristics of the participants in the China Multi-Ethnic Cohort (CMEC) follow-up survey

Variables	Overall (N=9,249)	Han Male <60 yrs (N=1,863)	Han Male ≥60 yrs (N=601)	Han Female < 60 yrs (N=2,689)	Han Female ≥60 yrs (N=522)	Minority Male <60 yrs (N=856)	Minority Male ≥60 yrs (N=277)	Minority Female <60 yrs (N=2,019)	Minority Female ≥60 yrs (N=422)	*p*-value[a]
MetS components										
**Waist circumference** (cm)	83.4±10.6	87.0±9.0	85.2±9.6	79.7±9.9	84.1±9.7	87.9±12.3	84.7±11.2	82.1±10.1	83.4±11.7	<0.001
**Triglycerides** (mmol/L)	1.7±1.4	2.1±1.8	1.5±1.1	1.5±1.2	1.6±1.1	1.9±1.9	1.5±0.9	1.5±1.1	1.6±1.0	<0.001
**HDL-C** (mmol/L)	1.4±0.4	1.2±0.3	1.4±0.4	1.4±0.3	1.5±0.4	1.3±0.3	1.4±0.4	1.5±0.4	1.5±0.4	<0.001
**Mean arterial pressure** (mmHg)	93.6±12.4	96.5±12.2	97.7±12.2	90.4±12.2	95.9±12.8	96.3±11.8	99.1±12.5	91.3±11.4	95.0±13.0	<0.001
**Fasting blood glucose** (mmol/L)	5.1±1.0	5.2±1.1	5.5±1.2	5.1±0.8	5.5±1.1	5.1±1.4	5.0±1.1	4.8±0.7	5.1±1.3	<0.001
CVD-related risk factors										
**Diabetes**										<0.001
No	8,438 (91.3)	1,679 (90.1)	506 (84.2)	2,533 (94.2)	432 (82.8)	767 (89.6)	235 (85.0)	1,916 (94.9)	370 (87.7)	
Yes	811 (8.7)	184 (9.9)	95 (15.8)	156 (5.8)	90 (17.2)	89 (10.4)	42 (15.0)	103 (5.1)	52 (12.3)	
**Hypertension**										<0.001
No	7,100 (76.8)	1,381 (74.1)	356 (59.2)	2,241 (83.3)	302 (57.9)	632 (73.8)	172 (62.1)	1,716 (85.0)	300 (71.1)	
Yes	2,149 (23.2)	482 (25.9)	245 (40.8)	448 (16.7)	220 (42.1)	224 (26.2)	105 (37.9)	303 (15.0)	122 (28.9)	
**Hyperlipidemia**										<0.001
No	6,201 (67.0)	1,068 (57.3)	408 (67.9)	1,962 (73.0)	322 (61.7)	476 (55.6)	191 (69.0)	1,506 (74.6)	268 (63.5)	
Yes	3,048 (33.0)	795 (42.7)	193 (32.1)	727 (27.0)	200 (38.3)	380 (44.4)	86 (31.0)	513 (25.4)	154 (36.5)	
CVD-related risk markers										
**HbA1c** (mmol/mol)	5.7±0.7	5.7±0.8	5.9±0.8	5.6±0.7	5.9±0.7	5.9±0.9	5.9±0.8	5.7±0.6	6.0±1.0	<0.001
**Total cholesterol** (mmol/L)	5.1±1.0	5.1±1.1	5.2±1.0	5.1±1.0	5.5±1.1	5.0±1.1	5.1±1.0	4.9±1.0	5.4±1.1	<0.001
**Body mass index** (kg/m^2)	24.6±3.6	25.1±3.5	24.2±3.0	24.1±3.6	24.7±3.4	25.2±3.7	23.9±3.4	24.7±3.8	23.9±4.0	0.096
**Serum uric acid** (μmol/L)	327.9±89.9	393.4±90.1	370.2±84.1	295.2±67.0	306.0±67.6	386.7±83.1	380.5±86.7	279.0±67.0	295.1±80.2	<0.001
Covariates										
**Marital status**										<0.001
Married	8,275 (89.4)	1,711 (91.9)	539 (89.7)	2,442 (90.8)	421 (80.7)	806 (94.2)	242 (87.4)	1,812 (89.7)	302 (71.6)	
Unmarried/divorced/widowed	974 (10.6)	152 (8.1)	62 (10.3)	247 (9.2)	101 (19.3)	50 (5.8)	35 (12.6)	207 (10.3)	120 (28.4)	
**Educational level**										<0.001

Percentage (%) or Mean±SD.

High school or above	2,490 (26.9)	883 (47.4)	116 (19.3)	1,057 (39.3)	71 (13.6)	126 (14.7)	45 (16.3)	173 (8.6)	19 (4.5)	
Primary and junior high school	4,530 (49.0)	906 (48.6)	402 (66.9)	1,391 (51.7)	259 (49.6)	485 (56.7)	130 (46.9)	871 (43.1)	86 (20.4)	
Illiteracy	2,229 (24.1)	74 (4.0)	83 (13.8)	241 (9.0)	192 (36.8)	245 (28.6)	102 (36.8)	975 (48.3)	317 (75.1)	
**Annual family income** (*yuan*)										<0.001
<12,000	1,375 (14.9)	134 (7.2)	114 (19.0)	208 (7.7)	89 (17.0)	156 (18.2)	81 (29.2)	448 (22.1)	145 (34.4)	
12,000-19,999	1,597 (17.2)	199 (10.7)	92 (15.3)	341 (12.7)	86 (16.5)	223 (26.1)	49 (17.7)	518 (25.7)	89 (21.1)	
20,000-59,999	3,334 (36.0)	654 (35.1)	211 (35.1)	999 (37.2)	186 (35.6)	336 (39.3)	89 (32.1)	739 (36.6)	120 (28.4)	
60,000-99,999	1,466 (15.9)	400 (21.5)	103 (17.1)	533 (19.8)	87 (16.7)	86 (10.0)	32 (11.6)	189 (9.4)	36 (8.5)	
≥100,000	1,477 (16.0)	476 (25.5)	81 (13.5)	608 (22.6)	74 (14.2)	55 (6.4)	26 (9.4)	125 (6.2)	32 (7.6)	
**Residential location**										<0.001
Urban	4,916 (53.2)	1,423 (76.4)	406 (67.6)	2,132 (79.3)	376 (72.0)	125 (14.6)	66 (23.8)	317 (15.7)	71 (16.8)	
Rural	4,333 (46.8)	440 (23.6)	195 (32.4)	557 (20.7)	146 (28.0)	731 (85.4)	211 (76.2)	1,702 (84.3)	351 (83.2)	
**Alcohol drinking**										<0.001
No	5,156 (55.7)	484 (26.0)	197 (32.8)	1,686 (62.7)	357 (68.4)	373 (43.6)	135 (48.7)	1,572 (77.9)	352 (83.4)	
Yes	4,093 (44.3)	1,379 (74.0)	404 (67.2)	1,003 (37.3)	165 (31.6)	483 (56.4)	142 (51.3)	447 (22.1)	70 (16.6)	
**Smoking**										<0.001
No	7,136 (77.2)	801 (43.0)	238 (39.6)	2,649 (98.5)	517 (99.0)	381 (44.5)	119 (43.0)	2,011 (99.6)	420 (99.5)	
Yes	2,113 (22.8)	1,062 (57.0)	363 (60.4)	40 (1.5)	5 (1.0)	475 (55.5)	158 (57.0)	8 (0.4)	2 (0.5)	
**Diet** (g/week)[b]										
Cereal	3,976.8±2,140.5	4,478.2±2,107.6	4,462.8±2,180.8	3,468.8±1,892.3	3,675.9±1,812.7	4,435.4±2,451.1	4,110.9±2,142.4	3,956.4±2,243.3	3,759.0±2,064.4	<0.001
Fruits	871.0±806.2	785.7±734.8	723.8±765.7	1,131.3±858.2	875.2±792.5	633.7±712.4	600.1±766.9	827.5±780.6	661.3±724.4	<0.001
Vegetables	2,206.9±1,455.8	2,184.9±1,489.7	2,734.2±1,702.5	2,237.5±1,457.0	2,426.9±1,554.8	2,171.6±1,410.2	1,989.5±1,303.1	2,079.5±1,353.1	1,909.3±1,214.1	<0.001
Meat	661.6±648.1	760.4±642.8	686.4±638.4	535.7±485.4	435.6±448.4	837.7±798.8	785.3±754.2	700.6±731.6	647.5±735.1	0.019
Soybean products	74.4±126.3	74.2±125.9	78.4±141.4	74.6±126.6	64.6±127.0	80.9±131.7	70.7±114.2	73.3±121.0	74.5±123.9	<0.079
Aquatic products	105.8±164.7	169.6±209.9	125.9±172.8	132.7±175.0	96.1±148.0	61.2±113.7	65.2±111.3	45.7±93.9	41.4±95.6	<0.001
**Physical activity** (METs/week)[c]	26.4±17.9	27.8±17.0	19.0±16.5	26.0±16.7	18.1±15.0	31.1±21.0	20.2±16.5	30.1±18.5	21.3±15.4	<0.001

CVD, cardiovascular disease; HbA1c, glycated haemoglobin; HDL-C, high-density lipoprotein cholesterol; METs, metabolic equivalent for the tasks; MetS, metabolic syndrome; SD, standard deviation.

[a]Significance of the differences in the values among the eight age-sex-ethnicity-specific subgroups, tested by the two-sided analysis of variance for continuous variables and the two-sided Chi-square test for categorical variable. [b]Diet was surveyed as how many grams were consumed on average per week during the preceding 12 months, measured by a mold of the standard serving size. [c]Physical activity was measured by the METs for occupational, traffic-related, chore, and leisure time activities on average per week during the preceding 12 months.

Table S4. Associations between the metabolic syndrome (MetS) score and the cardiovascular disease (CVD)-related risk factors and risk markers with the C-index in the China Multi-Ethnic Cohort (CMEC) follow-up survey

**Groups**	**HR (95% CI) and C-index (95% CI)**						
	**Hyperlipidemia**	**Diabetes**	**Hypertension**	**Elevated HbA1c**	**Elevated CHOL**	**Elevated BMI**	**Elevated SUA**
**Overall**[a]	1.72 (1.69, 1.75)[**]	1.61 (1.45, 1.78)[**]	1.42 (1.39, 1.45)[**]	1.63 (1.57, 1.68)[**]	1.41 (1.36, 1.46)[**]	1.68 (1.64, 1.72)[**]	1.58 (1.53, 1.63)[**]
C-index	0.80 (0.79, 0.80)	0.75 (0.73, 0.76)	0.71 (0.70, 0.72)	0.76 (0.75, 0.77)	0.65 (0.64, 0.66)	0.76 (0.75, 0.77)	0.75 (0.74, 0.76)
**Han**[b]							
**Male**							
<60	1.74 (1.68, 1.79)[**]	1.60 (1.39, 1.85)[**]	1.38 (1.32, 1.44)[**]	1.60 (1.48, 1.72)[**]	1.38 (1.27, 1.5)[**]	1.65 (1.57, 1.73)[**]	1.52 (1.42, 1.62)[**]
C-index	0.81 (0.80, 0.83)	0.73 (0.70, 0.76)	0.66 (0.64, 0.68)	0.73 (0.70, 0.76)	0.66 (0.63, 0.69)	0.75 (0.73, 0.77)	0.70 (0.67, 0.72)
≥60	2.06 (1.91, 2.22)[**]	1.49 (1.30, 1.70)[**]	1.16 (1.08, 1.24)[**]	1.46 (1.29, 1.65)[**]	1.43 (1.25, 1.63)[**]	1.94 (1.72, 2.20)[**]	1.42 (1.24, 1.62)[**]
C-index	0.83 (0.81, 0.86)	0.69 (0.64, 0.74)	0.59 (0.57, 0.62)	0.68 (0.64, 0.73)	0.73 (0.69, 0.77)	0.80 (0.76, 0.84)	0.67 (0.62, 0.72)
**Female**							
<60	2.06 (1.98, 2.15)[**]	1.86 (1.69, 2.05)[**]	1.51 (1.44, 1.59)[**]	1.95 (1.77, 2.14)[**]	1.48 (1.38, 1.59)[**]	1.72 (1.63, 1.82)[**]	1.78 (1.62, 1.94)[**]
C-index	0.81 (0.80, 0.83)	0.74 (0.71, 0.77)	0.70 (0.69, 0.72)	0.78 (0.75, 0.81)	0.67 (0.64, 0.69)	0.77 (0.76, 0.79)	0.73 (0.70, 0.77)
<60	1.72 (1.56, 1.88)[**]	1.41 (1.19, 1.65)[**]	1.12 (1.03, 1.22)[*]	1.37 (1.17, 1.61)[**]	1.04 (0.91, 1.19)	1.58 (1.38, 1.81)[**]	1.60 (1.33, 1.92)[**]
C-index	0.71 (0.68, 0.75)	0.68 (0.62, 0.73)	0.60 (0.57, 0.63)	0.68 (0.63, 0.73)	0.61 (0.56, 0.65)	0.69 (0.65, 0.73)	0.69 (0.64, 0.75)
**Minority**[b]							
**Male**							
<60	2.05 (1.91, 2.21)[**]	1.88 (1.53, 2.31)[**]	1.79 (1.63, 1.96)[**]	1.80 (1.49, 2.17)[**]	1.58 (1.38, 1.81)[**]	2.66 (2.36, 2.99)[**]	1.84 (1.63, 2.09)[**]
C-index	0.77 (0.75, 0.79)	0.76 (0.71, 0.81)	0.71 (0.69, 0.74)	0.77 (0.73, 0.82)	0.66 (0.62, 0.70)	0.83 (0.81, 0.86)	0.76 (0.72, 0.79)
≥60	2.13 (1.8, 2.53)[**]	2.04 (1.40, 2.96)[**]	1.40 (1.21, 1.61)[**]	1.72 (1.30, 2.26)[**]	1.41 (1.08, 1.84)[*]	3.25 (2.30, 4.60)[**]	1.64 (1.27, 2.11)[**]
C-index	0.77 (0.73, 0.82)	0.83 (0.77, 0.89)	0.65 (0.61, 0.70)	0.72 (0.65, 0.80)	0.77 (0.70, 0.83)	0.90 (0.86, 0.93)	0.78 (0.71, 0.84)
**Female**							
<60	2.02 (1.93, 2.13)[**]	1.88 (1.66, 2.13)[**]	1.56 (1.46, 1.67)[**]	1.90 (1.69, 2.14)[**]	1.53 (1.41, 1.67)[**]	1.53 (1.43, 1.64)[**]	1.79 (1.60, 2.01)[**]
C-index	0.78 (0.76, 0.80)	0.77 (0.73, 0.82)	0.68 (0.66, 0.71)	0.77 (0.73, 0.81)	0.66 (0.63, 0.69)	0.69 (0.67, 0.72)	0.76 (0.72, 0.79)
≥60	1.54 (1.41, 1.68)[**]	1.53 (1.30, 1.80)[**]	1.26 (1.14, 1.39)[**]	1.48 (1.27, 1.73)[**]	1.12 (0.98, 1.28)	1.56 (1.35, 1.79)[**]	1.67 (1.38, 2.02)[**]
C-index	0.68 (0.64, 0.73)	0.72 (0.65, 0.78)	0.64 (0.60, 0.68)	0.73 (0.68, 0.79)	0.62 (0.58, 0.67)	0.79 (0.75, 0.83)	0.81 (0.75, 0.86)

BMI, body mass index; CHOL, cholesterol; CI, confidence interval; HbA1c, glycated hemoglobin; HR: hazard ratios; SUA, serum uric acid.

[a]Adjusted for age, sex, ethnicity, marital status, educational level, annual family income, residential location, alcohol drinking status, smoking status, diet, and physical activity at baseline.

[b]Adjusted for marital status, educational level, annual family income, residential location, alcohol drinking status, smoking status, diet, and physical activity at baseline.

HR is estimated by Cox regression, and all tests are two-sided. [*]$p<0.05$, [**]$p<0.001$

Table S5. Capacities of the traditionally defined metabolic syndrome (MetS) and the dichotomous age-sex-ethnicity-specific MetS score in detecting one or more cardiovascular disease (CVD)-related risk factors and risk markers in the China Multi-Ethnic Cohort (CMEC) dataset

**Groups**	**MetS prevalence (95% CI)**		**Sensitivity (95% CI)**		**Specificity (95% CI)**		**AUC (95% CI)**	
	Traditional	Dichotomous	Traditional	Dichotomous	Traditional	Dichotomous	Traditional	Dichotomous
			**Detecting one or more CVD-related risk factors**					
**Overall**	0.19 (0.19, 0.20)	0.30 (0.30, 0.30)*	0.37 (0.37, 0.38)	0.53 (0.53, 0.54)*	0.95 (0.94, 0.95)	0.90 (0.89, 0.90)*	0.66 (0.66, 0.66)	0.71 (0.71, 0.72)*
**Han**								
**Male**								
<60	0.24 (0.23, 0.25)	0.35 (0.34, 0.36)*	0.42 (0.40, 0.43)	0.61 (0.60, 0.62)*	0.95 (0.95, 0.96)	0.93 (0.93, 0.94)*	0.68 (0.68, 0.69)	0.77 (0.76, 0.78)*
≥60	0.20 (0.19, 0.21)	0.31 (0.30, 0.32)*	0.31 (0.30, 0.33)	0.43 (0.42, 0.45)*	0.95 (0.94, 0.96)	0.86 (0.85, 0.88)*	0.63 (0.62, 0.64)	0.65 (0.64, 0.66)*
**Female**								
<60	0.13 (0.12, 0.13)	0.23 (0.23, 0.24)*	0.33 (0.32, 0.34)	0.55 (0.54, 0.56)*	0.96 (0.96, 0.96)	0.90 (0.90, 0.91)*	0.64 (0.64, 0.65)	0.73 (0.72, 0.73)*
≥60	0.30 (0.29, 0.31)	0.41 (0.40, 0.42)*	0.42 (0.40, 0.44)	0.52 (0.50, 0.54)*	0.87 (0.86, 0.89)	0.74 (0.73, 0.76)*	0.65 (0.63, 0.66)	0.63 (0.62, 0.65)*
**Minority**								
**Male**								
<60	0.24 (0.23, 0.25)	0.33 (0.32, 0.34)*	0.40 (0.38, 0.41)	0.53 (0.52, 0.55)*	0.95 (0.95, 0.96)	0.92 (0.91, 0.93)*	0.67 (0.67, 0.68)	0.73 (0.72, 0.74)*
≥60	0.19 (0.18, 0.21)	0.34 (0.32, 0.36)*	0.29 (0.27, 0.31)	0.47 (0.45, 0.49)*	0.95 (0.94, 0.97)	0.86 (0.84, 0.88)*	0.62 (0.61, 0.63)	0.67 (0.65, 0.68)*
**Female**								
<60	0.17 (0.16, 0.17)	0.24 (0.24, 0.25)*	0.37 (0.36, 0.38)	0.52 (0.51, 0.53)*	0.94 (0.94, 0.95)	0.90 (0.90, 0.91)*	0.65 (0.65, 0.66)	0.71 (0.71, 0.72)*
≥60	0.25 (0.24, 0.27)	0.31 (0.29, 0.32)*	0.37 (0.35, 0.39)	0.42 (0.40, 0.44)*	0.91 (0.90, 0.93)	0.85 (0.84, 0.87)*	0.64 (0.63, 0.66)	0.64 (0.63, 0.65)
			**Detecting one or more CVD-related risk markers**					
**Overall**			0.37 (0.36, 0.38)	0.49 (0.49, 0.50)*	0.90 (0.89, 0.90)	0.81 (0.81, 0.82)*	0.63 (0.63, 0.64)	0.65 (0.65, 0.66)*
**Han**								
**Male**								
<60			0.41 (0.40, 0.42)	0.55 (0.54, 0.56)*	0.89 (0.88, 0.90)	0.80 (0.79, 0.81)*	0.65 (0.64, 0.66)	0.67 (0.67, 0.68)*
≥60			0.36 (0.33, 0.38)	0.48 (0.46, 0.51)*	0.89 (0.88, 0.90)	0.79 (0.78, 0.81)*	0.62 (0.61, 0.63)	0.64 (0.62, 0.65)*
**Female**								
<60			0.31 (0.30, 0.32)	0.45 (0.44, 0.47)*	0.93 (0.92, 0.93)	0.84 (0.83, 0.84)*	0.62 (0.61, 0.63)	0.65 (0.64, 0.65)*
≥60			0.45 (0.43, 0.47)	0.54 (0.52, 0.56)*	0.80 (0.79, 0.82)	0.68 (0.66, 0.70)*	0.63 (0.61, 0.64)	0.61 (0.60, 0.63)*
**Minority**								
**Male**								
<60			0.38 (0.37, 0.40)	0.52 (0.50, 0.53)*	0.89 (0.88, 0.89)	0.83 (0.82, 0.84)*	0.63 (0.62, 0.64)	0.67 (0.66, 0.68)*
≥60			0.33 (0.30, 0.36)	0.50 (0.47, 0.53)*	0.91 (0.90, 0.93)	0.79 (0.77, 0.81)*	0.62 (0.61, 0.64)	0.65 (0.63, 0.66)*
**Female**								
<60			0.34 (0.33, 0.36)	0.44 (0.43, 0.45)*	0.90 (0.90, 0.91)	0.83 (0.83, 0.84)*	0.62 (0.62, 0.63)	0.64 (0.63, 0.65)*

≥60	0.39 (0.37, 0.42)	0.43 (0.40, 0.45)*	0.84 (0.83, 0.86)	0.78 (0.76, 0.79)*	0.62 (0.60, 0.63)	0.60 (0.59, 0.62)

AUC, area under the curve; CI: confidence interval. **p*<0.05 when comparing the traditionally defined MetS and the dichotomous age-sex-ethnicity-specific MetS score.

Table S6. Characteristics of the participants in the Yunnan Behavior and Disease Surveillance (YBDS) survey

Variables	Overall (N=39,065)	Han				Minority				*p*-value[a]
		Male		Female		Male		Female		
		<60 yrs	≥60 yrs	< 60 yrs	≥60 yrs	<60 yrs	≥60 yrs	<60 yrs	≥60 yrs	
		(N=6,418)	(N=3,800)	(N=8,575)	(N=3,891)	(N=5,392)	(N=1,940)	(N=6,724)	(N=2,325)	
MetS components										
**Waist circumference** (cm)	81.1±9.7	82.7±9.5	80.8±9.3	79.7±9.3	80.4±9.8	83.0±9.5	81.3±9.5	80.3±9.8	80.9±10.2	<0.001
**Triglycerides** (mmol/L)	1.9±1.5	2.1±1.8	1.6±1.2	1.7±1.3	1.8±1.3	2.3±1.9	1.8±1.5	1.8±1.5	2.0±1.5	<0.001
**HDL-C** (mmol/L)	1.5±0.5	1.3±0.4	1.5±0.4	1.4±0.3	1.5±0.4	1.5±0.7	1.6±0.6	1.5±0.6	1.6±0.6	<0.001
**Mean arterial pressure** (mmHg)	95.6±13.0	96.0±12.4	97.6±13.3	92.7±12.3	96.8±13.2	97.3±12.7	100.5±14.7	93.2±12.5	98.4±13.6	<0.001
**Fasting blood glucose** (mmol/L)	5.2±1.1	5.2±1.2	5.3±1.2	5.1±1.1	5.3±1.2	5.3±1.2	5.4±1.2	5.2±1.0	5.4±1.2	<0.001
CVD-related risk factors										
**Diabetes**										<0.001
No	34,573 (88.5)	5,840 (91.0)	3,252 (85.6)	7,913 (92.3)	3,330 (85.6)	4,710 (87.4)	1,570 (80.9)	6,021 (89.5)	1,937 (83.3)	
Yes	4,492 (11.5)	578 (9.0)	548 (14.4)	662 (7.7)	561 (14.4)	682 (12.6)	370 (19.1)	703 (10.5)	388 (16.7)	
**Hypertension**										<0.001
No	27,822 (71.2)	4,824 (75.2)	2,296 (60.4)	6,858 (80.0)	2,260 (58.1)	3,876 (71.9)	1,041 (53.7)	5,339 (79.4)	1,328 (57.1)	
Yes	11,243 (28.8)	1,594 (24.8)	1,504 (39.6)	1,717 (20.0)	1,631 (41.9)	1,516 (28.1)	899 (46.3)	1,385 (20.6)	997 (42.9)	
**Hyperlipidemia**										<0.001
No	25,620 (65.6)	3,815 (59.4)	2,703 (71.1)	6,177 (72.0)	2,530 (65.0)	2,951 (54.7)	1,294 (66.7)	4,695 (69.8)	1,455 (62.6)	
Yes	13,445 (34.4)	2,603 (40.6)	1,097 (28.9)	2,398 (28.0)	1,361 (35.0)	2,441 (45.3)	646 (33.3)	2,029 (30.2)	870 (37.4)	
CVD-related risk markers										
**HbA1c** (mmol/mol)	5.8±0.8	5.7±0.8	5.9±0.8	5.7±0.7	6.0±0.8	5.7±0.8	5.9±0.8	5.7±0.8	5.9±0.8	<0.001
**Total cholesterol** (mmol/L)	5.1±1.1	5.1±1.0	5.0±1.0	4.9±1.0	5.4±1.0	5.2±1.2	5.1±1.1	5.0±1.2	5.5±1.2	<0.001
**Body mass index** (kg/m^2)	23.9±3.9	24.1±4.3	22.7±3.2	24.3±3.7	23.3±3.5	24.1±3.7	22.9±3.4	24.6±4.2	23.3±3.8	<0.001
**Serum uric acid** (μmol/L)	317.0±87.5	365.7±83.5	355.6±84.8	274.1±64.7	282.7±72.2	372.8±85.5	363.0±84.1	275.9±67.8	285.9±69.4	<0.001
Covariates										
**Marital status**										<0.001
Married	35,999 (92.1)	5,981 (93.2)	3,510 (92.4)	8,311 (96.9)	3,150 (81.1)	4,865 (90.2)	1,798 (92.6)	6,492 (96.6)	1,892 (81.4)	
Unmarried/divorced/widowed	3,066 (7.9)	437 (6.8)	290 (7.7)	264 (3.1)	741 (19.1)	527 (9.8)	142 (7.4)	232 (3.4)	433 (18.6)	
**Educational level**										<0.001

Percentage (%) or Mean±SD

High school or above	2,710 (6.9)	614 (9.6)	234 (6.2)	753 (8.8)	135 (3.5)	399 (7.4)	123 (6.3)	416 (6.2)	36 (1.5)	
Primary and junior high school	28,102 (72.0)	5,481 (85.4)	2,903 (76.4)	4,375 (74.3)	1,713 (44.0)	4,371 (81.1)	1,398 (72.1)	4,726 (70.3)	1,135 (48.8)	
Illiteracy	8,253 (21.1)	323 (5.0)	663 (17.4)	1,447 (16.9)	2,043 (52.5)	622 (11.5)	419 (21.6)	1,582 (23.5)	1,154 (49.7)	
**Medical insurance**										<0.001
No	312 (0.8)	55 (0.9)	51 (1.3)	83 (1.0)	49 (1.3)	25 (0.5)	8 (0.4)	28 (0.4)	13 (0.6)	
Yes	38,753 (99.2)	6,363 (99.1)	3,749 (98.7)	8,492 (99.0)	3,842 (98.7)	5,367 (99.5)	1,932 (99.6)	6,696 (99.6)	2,312 (99.4)	
**Alcohol drinking**										<0.001
No	26,290 (67.3)	2,451 (38.2)	1,939 (51.0)	7,585 (88.5)	3,565 (91.6)	2,109 (39.1)	989 (51.0)	5,588 (83.1)	2,064 (88.8)	
Yes	12,775 (32.7)	3,967 (61.8)	1,861 (49.0)	990 (11.5)	326 (8.4)	3,283 (60.9)	951 (49.0)	1,136 (16.9)	261 (11.2)	
**Smoking**										<0.001
No	25,920 (66.4)	1,439 (22.4)	895 (23.6)	8,515 (99.3)	3,841 (98.8)	1,779 (33.0)	694 (35.8)	6,538 (97.2)	2,219 (95.4)	
Yes	13,145 (33.6)	4,979 (77.6)	2,905 (73.4)	60 (0.7)	50 (1.2)	3,613 (67.0)	1,246 (64.2)	186 (2.8)	106 (4.6)	
**Diet** (g/week)[b]										
Cereal	2,795.0±3,162.8	3,146.2±2,896.2	3,070.5±4,413.8	2,530.1±2,429.7	2,516.6±3,785.9	3,219.6±3,628.9	2,820.8±2,854.5	2,685.7±2,798.4	2,286.9±2,046.5	<0.001
Fruits	943.7±1,703.7	898.8±2,115.6	871.8±1,413.1	1,136.8±1,805.5	947.1±1,936.4	786.7±1,407.7	752.6±1,147.9	972.9±1,462.9	843.0±1,669.9	<0.001
Vegetables	2,605.3±2,371.8	2,782.8±2,353.2	2,872.8±2,613.7	2,743.9±2,295.0	2,601.0±2,360.7	2,471.1±2,485.9	2,372.5±2,178.1	2,424.9±2,418.1	2,236.4±2,046.5	<0.001
Meat	1,094.8±1,521.5	1,252.8±1,623.1	1,053.9±1,570.7	957.7±1,432.7	771.7±1,099.5	1,410.2±1,814.3	1,170.9±1,372.2	1,148.6±1,574.8	987.9±1,339.0	<0.001
**Physical activity**[c]										<0.001
<4 days/week	11,845 (30.3)	2,475 (38.6)	1,323 (34.8)	2,391 (27.9)	1,114 (28.6)	1,864 (34.6)	592 (30.5)	1,543 (22.9)	543 (23.4)	
≥4 days/week	27,220 (69.7)	3,943 (61.4)	2,477 (65.2)	6,184 (72.1)	2,777 (71.4)	3,528 (65.4)	1,348 (69.5)	5,181 (77.1)	1,782 (76.6)	

CVD, cardiovascular disease; HbA1c, glycated haemoglobin; HDL-C, high-density lipoprotein cholesterol; MetS, metabolic syndrome; SD, standard deviation.
[a]Significance of the differences in the values among the eight age-sex-ethnicity-specific subgroups, tested by the two-sided analysis of variance for continuous variables and the two-sided Chi-square test for categorical variable. [b]Diet was surveyed as how many grams were consumed on average per week during the preceding 12 months, measured by a mold of the standard serving size. [c]Physical activity was measured by asking the question "In your work, farm work, and household activities, how many days per week on average do you have moderate- to high-intensity activities that cause a slight increase in respiration and heart rate?".

Table S7. Associations between the metabolic syndrome (MetS) score and the cardiovascular disease (CVD)-related risk factors and risk markers in the Yunnan Behavior and Disease Surveillance (YBDS) survey

Groups	OR (95% CI)			β (95% CI)			
	Hyperlipidemia	Diabetes	Hypertension	HbA1c	CHOL	BMI	SUA
**Overall**[a]	1.31 (1.31, 1.32)**	1.08 (1.07, 1.08)**	1.14 (1.13, 1.14)**	0.22 (0.21, 0.23)**	0.19 (0.18, 0.20)**	2.11 (2.07, 2.15)**	24.57 (23.65-25.48)**
**Han**[b]							
**Male**							
<60	1.38 (1.36, 1.40)**	1.07 (1.06, 1.08)**	1.11 (1.10, 1.13)**	0.19 (0.17, 0.22)**	0.23 (0.20, 0.26)**	2.13 (2.03, 2.23)**	26.04 (23.53, 28.55)**
≥60	1.28 (1.26, 1.30)**	1.06 (1.04, 1.07)**	1.09 (1.07, 1.11)**	0.15 (0.12, 0.18)**	0.12 (0.08, 0.15)**	1.61 (1.50, 1.71)**	20.34 (17.20, 23.48)**
**Female**							
<60	1.31 (1.30, 1.33)**	1.07 (1.06, 1.08)**	1.11 (1.10, 1.12)**	0.21 (0.20, 0.23)**	0.19 (0.17, 0.21)**	1.73 (1.65, 1.81)**	21.09 (19.58, 22.6)**
≥60	1.26 (1.24, 1.28)**	1.06 (1.04, 1.07)**	1.07 (1.05, 1.09)**	0.14 (0.11, 0.17)**	0.14 (0.10, 0.18)**	1.34 (1.22, 1.46)**	19.88 (17.35, 22.4)**
**Minority**[b]							
**Male**							
<60	1.23 (1.21, 1.25)**	1.06 (1.05, 1.08)**	1.14 (1.12, 1.16)**	0.21 (0.18, 0.23)**	0.20 (0.16, 0.23)**	2.39 (2.29, 2.48)**	27.13 (24.24, 30.03)**
≥60	1.21 (1.17, 1.23)**	1.07 (1.04, 1.09)**	1.14 (1.11, 1.18)**	0.16 (0.12, 0.21)**	0.00 (-0.06, 0.06)	2.02 (1.85, 2.19)**	18.98 (14.02, 23.94)**
**Female**							
<60	1.35 (1.33, 1.36)**	1.08 (1.07, 1.09)**	1.14 (1.13, 1.15)**	0.21 (0.19, 0.23)**	0.23 (0.20, 0.26)**	1.97 (1.86, 2.08)**	23.37 (21.37, 25.36)**
≥60	1.21 (1.19, 1.24)**	1.07 (1.05, 1.08)**	1.10 (1.07, 1.12)**	0.18 (0.15, 0.22)**	0.13 (0.09, 0.18)**	1.55 (1.39, 1.71)**	18.58 (15.42, 21.74)**

BMI, body mass index; CHOL, cholesterol; CI, confidence interval; HbA1c, glycated hemoglobin; OR, odds ratio; SUA, serum uric acid.

[a]Adjusted for age, sex, ethnicity, marital status, educational level, medical insurance, alcohol drinking, smoking, diet, and physical activity. [b]Adjusted for marital status, educational level, medical insurance, alcohol drinking, smoking, diet, and physical activity.

OR and β are estimated by multiple logistic regression and linear regression, respectively, and all tests are two-sided. $^{*}p<0.05$, $^{**}p<0.001$

Table S8. Associations between the metabolic syndrome (MetS) score in quartiles and the cardiovascular disease (CVD)-related risk factors and risk markers in the Yunnan Behavior and Disease Surveillance (YBDS) survey

Groups	Quartiles	OR (95% CI)						
		**Hyperlipidemia**	**Diabetes**	**Hypertension**	**Elevated HbA1c**	**Elevated CHOL**	**Elevated BMI**	**Elevated SUA**
**Overall**[a]	<-0.48	1.00	1.00	1.00	1.00	1.00	1.00	1.00
	[-0.48, -0.01)	1.04 (1.02, 1.05)**	1.01 (1.00, 1.02)*	1.08 (1.07, 1.10)**	1.01 (1.00, 1.02)*	1.00 (0.99, 1.01)	1.03 (1.02, 1.04)**	1.03 (1.02, 1.04)**
	[-0.01, 0.57)	1.19 (1.18, 1.21)**	1.05 (1.04, 1.06)**	1.19 (1.17, 1.21)**	1.04 (1.03, 1.05)**	1.02 (1.00, 1.03)*	1.13 (1.12, 1.14)**	1.10 (1.09, 1.12)**
	>0.57	1.89 (1.87, 1.92)**	1.16 (1.15, 1.17)**	1.35 (1.34, 1.37)**	1.14 (1.13, 1.15)**	1.09 (1.08, 1.10)**	1.36 (1.34, 1.37)**	1.25 (1.23, 1.26)**
**Han**[b]								
**Male**								
<60	<-0.67	1.00	1.00	1.00	1.00	1.00	1.00	1.00
	[-0.67, -0.15)	1.10 (1.07, 1.14)**	0.99 (0.97, 1.01)	1.05 (1.01, 1.09)*	1.00 (0.98, 1.02)	1.02 (0.99, 1.05)	1.01 (0.99, 1.04)	1.04 (1.01, 1.08)*
	[-0.15, 0.44)	1.45 (1.41, 1.50)**	1.05 (1.03, 1.08)**	1.15 (1.11, 1.19)**	1.04 (1.02, 1.06)**	1.06 (1.03, 1.09)**	1.13 (1.10, 1.16)**	1.15 (1.11, 1.19)**
	≥0.44	2.27 (2.20, 2.35)**	1.15 (1.12, 1.17)**	1.30 (1.26, 1.35)**	1.14 (1.12, 1.16)**	1.13 (1.10, 1.16)**	1.39 (1.35, 1.43)**	1.31 (1.27, 1.36)**
≥60	<-0.62	1.00	1.00	1.00	1.00	1.00	1.00	1.00
	[-0.62, -0.11)	0.99 (0.95, 1.04)	1.00 (0.96, 1.04)	1.08 (1.02, 1.14)*	1.01 (0.98, 1.04)	0.96 (0.93, 1.00)*	1.00 (0.98, 1.03)	1.04 (0.99, 1.08)
	[-0.11, 0.50)	1.12 (1.07, 1.16)**	1.06 (1.02, 1.10)*	1.12 (1.06, 1.18)**	1.06 (1.03, 1.10)**	0.99 (0.96, 1.02)	1.02 (1.00, 1.05)	1.08 (1.04, 1.13)**
	≥0.50	1.82 (1.74, 1.90)**	1.13 (1.09, 1.17)**	1.23 (1.16, 1.30)**	1.14 (1.10, 1.17)**	1.02 (0.99, 1.06)	1.17 (1.14, 1.20)**	1.26 (1.21, 1.32)**
**Female**								
<60	<-0.29	1.00	1.00	1.00	1.00	1.00	1.00	1.00
	[-0.29, 0.18)	1.02 (0.99, 1.04)	1.02 (1.00, 1.04)*	1.06 (1.03, 1.08)**	1.01 (1.00, 1.03)	1.00 (0.98, 1.02)	1.05 (1.03, 1.07)**	1.02 (1.00, 1.04)*
	[0.18, 0.74)	1.14 (1.11, 1.16)**	1.04 (1.03, 1.06)**	1.16 (1.13, 1.19)**	1.04 (1.02, 1.05)**	1.02 (1.00, 1.04)	1.17 (1.15, 1.20)**	1.09 (1.07, 1.11)**
	≥0.74	1.97 (1.92, 2.01)**	1.14 (1.12, 1.16)**	1.32 (1.29, 1.36)**	1.12 (1.11, 1.14)**	1.08 (1.06, 1.10)**	1.36 (1.33, 1.39)**	1.18 (1.15, 1.20)**
≥60	<-0.42	1.00	1.00	1.00	1.00	1.00	1.00	1.00
	[-0.42, 0.12)	0.96 (0.92, 1.00)	1.00 (0.97, 1.04)	1.06 (1.00, 1.11)*	1.01 (0.98, 1.04)	0.96 (0.93, 1.00)	1.02 (0.99, 1.05)	1.03 (1.00, 1.07)
	[0.12, 0.68)	1.03 (0.99, 1.07)	1.06 (1.02, 1.09)*	1.11 (1.05, 1.17)**	1.05 (1.02, 1.08)*	0.96 (0.92, 1.00)	1.09 (1.06, 1.13)**	1.08 (1.04, 1.12)**
	≥0.68	1.78 (1.71, 1.86)**	1.14 (1.10, 1.17)**	1.19 (1.13, 1.25)**	1.13 (1.10, 1.17)**	1.05 (1.01, 1.10)*	1.18 (1.14, 1.21)**	1.19 (1.15, 1.23)**
**Minority**[b]								
**Male**								
<60	<-0.83	1.00	1.00	1.00	1.00	1.00	1.00	1.00
	[-0.83, -0.26)	1.02 (0.98, 1.06)	1.02 (0.99, 1.05)	1.08 (1.04, 1.13)**	1.02 (1.00, 1.05)	1.01 (0.98, 1.04)	1.02 (0.99, 1.05)	1.02 (0.98, 1.07)
	[-0.26, 0.38)	1.21 (1.16, 1.25)**	1.06 (1.03, 1.09)**	1.13 (1.09, 1.18)**	1.03 (1.01, 1.06)*	1.03 (1.00, 1.06)	1.08 (1.05, 1.11)**	1.11 (1.07, 1.16)**
	≥0.38	1.70 (1.63, 1.77)**	1.13 (1.10, 1.17)**	1.36 (1.31, 1.41)**	1.12 (1.09, 1.15)**	1.12 (1.09, 1.16)**	1.48 (1.44, 1.52)**	1.31 (1.26, 1.37)**
≥60	<-0.61	1.00	1.00	1.00	1.00	1.00	1.00	1.00
	[-0.61, -0.04)	0.98 (0.92, 1.05)	1.00 (0.94, 1.06)	1.05 (0.98, 1.14)	1.01 (0.96, 1.07)	0.98 (0.93, 1.03)	0.98 (0.95, 1.02)	0.98 (0.91, 1.04)
	[-0.04, 0.53)	1.07 (1.00, 1.14)	1.08 (1.02, 1.15)*	1.21 (1.12, 1.31)**	1.06 (1.01, 1.11)*	0.96 (0.92, 1.01)	1.04 (1.00, 1.07)	1.08 (1.02, 1.16)*

	≥0.53	1.52 (1.42, 1.63)**	1.13 (1.06, 1.20)**	1.35 (1.25, 1.46)**	1.13 (1.07, 1.19)**	1.01 (0.96, 1.06)	1.20 (1.16, 1.25)**	1.20 (1.12, 1.28)**
**Female**								
<60	<-0.39	1.00	1.00	1.00	1.00	1.00	1.00	1.00
	[-0.39, 0.05)	1.02 (0.99, 1.04)	1.00 (0.98, 1.02)	1.09 (1.06, 1.12)**	0.99 (0.97, 1.01)	1.00 (0.98, 1.02)	1.06 (1.04, 1.09)**	1.03 (1.01, 1.05)*
	[0.05, 0.66)	1.18 (1.15, 1.21)**	1.03 (1.00, 1.05)*	1.20 (1.16, 1.23)**	1.01 (0.99, 1.04)	1.03 (1.00, 1.05)*	1.22 (1.19, 1.26)**	1.09 (1.07, 1.12)**
	≥0.66	2.03 (1.98, 2.09)**	1.17 (1.14, 1.19)**	1.35 (1.31, 1.39)**	1.13 (1.10, 1.15)**	1.10 (1.08, 1.12)**	1.39 (1.35, 1.42)**	1.20 (1.17, 1.23)**
≥60	<-0.45	1.00	1.00	1.00	1.00	1.00	1.00	1.00
	[-0.45, 0.11)	0.94 (0.89, 1.00)	1.01 (0.97, 1.06)	1.07 (1.01, 1.14)*	1.00 (0.96, 1.05)	0.96 (0.91, 1.01)	1.03 (0.99, 1.08)	1.04 (1.00, 1.10)
	[0.11, 0.73)	1.04 (0.98, 1.10)	1.05 (1.01, 1.10)*	1.22 (1.14, 1.30)**	1.08 (1.03, 1.12)**	1.03 (0.97, 1.08)	1.12 (1.08, 1.16)**	1.12 (1.07, 1.18)**
	≥0.73	1.60 (1.51, 1.69)**	1.16 (1.11, 1.22)**	1.28 (1.20, 1.37)**	1.15 (1.10, 1.20)**	1.07 (1.01, 1.13)*	1.24 (1.20, 1.29)**	1.24 (1.18, 1.30)**

BMI, body mass index; CHOL, cholesterol; CI, confidence interval; HbA1c, glycated hemoglobin; OR: odds ratios; SUA, serum uric acid.

[a]Adjusted for age, sex, ethnicity, marital status, educational level, medical insurance, alcohol drinking, smoking, diet, and physical activity. [b]Adjusted for marital status, educational level, medical insurance, alcohol drinking, smoking, diet, and physical activity.

OR is estimated by multiple logistic regression, and all tests are two-sided. $^{*}p<0.05$, $^{**}p<0.001$

Table S9. Capacities of the traditionally defined metabolic syndrome (MetS) and the dichotomous age-sex-ethnicity-specific MetS in detecting one or more cardiovascular disease (CVD)-related risk factors in the Yunnan Behavior and Disease Surveillance (YBDS) survey

**Groups**	**MetS prevalence (95% CI)**		**Sensitivity (95% CI)**		**Specificity (95% CI)**		**AUC (95% CI)**	
	Traditional	Dichotomous	Traditional	Dichotomous	Traditional	Dichotomous	Traditional	Dichotomous
			**Detecting one or more CVD-related risk factors**					
**Overall**	0.23 (0.23, 0.24)	0.31 (0.31, 0.31)*	0.38 (0.37, 0.39)	0.50 (0.49, 0.50)*	0.94 (0.94, 0.95)	0.92 (0.92, 0.93)*	0.66 (0.66, 0.66)	0.71 (0.70, 0.71)*
**Han**								
**Male**								
<60	0.23 (0.22, 0.24)	0.30 (0.29, 0.31)*	0.38 (0.36, 0.40)	0.51 (0.50, 0.53)*	0.97 (0.96, 0.97)	0.96 (0.95, 0.97)	0.67 (0.66, 0.68)	0.74 (0.73, 0.75)*
≥60	0.17 (0.16, 0.18)	0.28 (0.27, 0.30)*	0.26 (0.25, 0.28)	0.41 (0.39, 0.43)*	0.97 (0.96, 0.98)	0.92 (0.91, 0.93)*	0.62 (0.61, 0.63)	0.67 (0.66, 0.68)*
**Female**								
<60	0.23 (0.22, 0.24)	0.30 (0.29, 0.31)*	0.44 (0.42, 0.45)	0.58 (0.57, 0.60)*	0.93 (0.92, 0.94)	0.92 (0.92, 0.93)*	0.68 (0.67, 0.69)	0.75 (0.74, 0.76)*
≥60	0.31 (0.29, 0.32)	0.49 (0.47, 0.50)*	0.42 (0.40, 0.44)	0.59 (0.57, 0.61)*	0.90 (0.88, 0.92)	0.70 (0.68, 0.73)*	0.66 (0.65, 0.67)	0.65 (0.63, 0.66)
**Minority**								
**Male**								
<60	0.23 (0.22, 0.25)	0.24 (0.23, 0.25)*	0.36 (0.34, 0.38)	0.37 (0.36, 0.39)*	0.97 (0.96, 0.97)	0.96 (0.96, 0.97)	0.66 (0.65, 0.67)	0.67 (0.66, 0.68)
≥60	0.20 (0.18, 0.22)	0.32 (0.30, 0.34)*	0.28 (0.26, 0.31)	0.43 (0.40, 0.46)*	0.98 (0.96, 0.99)	0.91 (0.88, 0.93)*	0.63 (0.62, 0.64)	0.67 (0.65, 0.69)*
**Female**								
<60	0.22 (0.21, 0.23)	0.26 (0.25, 0.27)*	0.39 (0.38, 0.41)	0.52 (0.50, 0.54)*	0.93 (0.92, 0.94)	0.97 (0.96, 0.97)*	0.66 (0.65, 0.67)	0.75 (0.74, 0.75)*
≥60	0.33 (0.31, 0.35)	0.36 (0.34, 0.38)*	0.44 (0.41, 0.46)	0.47 (0.44, 0.49)*	0.90 (0.88, 0.93)	0.85 (0.82, 0.88)*	0.67 (0.65, 0.69)	0.66 (0.64, 0.68)
			**Detecting one or more CVD-related risk markers**					
**Overall**			0.37 (0.36, 0.38)	0.47 (0.46, 0.48)*	0.84 (0.83, 0.84)	0.77 (0.77, 0.78)*	0.60 (0.60, 0.61)	0.62 (0.61, 0.62)*
**Han**								
**Male**								
<60			0.37 (0.35, 0.39)	0.48 (0.46, 0.50)*	0.86 (0.84, 0.87)	0.80 (0.78, 0.81)*	0.61 (0.60, 0.62)	0.64 (0.63, 0.65)*
≥60			0.29 (0.26, 0.31)	0.43 (0.41, 0.46)*	0.89 (0.88, 0.91)	0.80 (0.79, 0.82)*	0.59 (0.58, 0.60)	0.62 (0.60, 0.63)*
**Female**								
<60			0.42 (0.39, 0.44)	0.51 (0.49, 0.53)*	0.83 (0.82, 0.84)	0.77 (0.76, 0.78)*	0.62 (0.61, 0.63)	0.64 (0.63, 0.65)*
≥60			0.44 (0.42, 0.47)	0.59 (0.57, 0.62)*	0.77 (0.75, 0.78)	0.57 (0.55, 0.59)*	0.61 (0.59, 0.62)	0.58 (0.56, 0.60)*
**Minority**								
**Male**								
<60			0.34 (0.32, 0.36)	0.40 (0.37, 0.42)*	0.85 (0.84, 0.87)	0.83 (0.82, 0.85)*	0.60 (0.58, 0.61)	0.61 (0.60, 0.63)*
≥60			0.30 (0.26, 0.33)	0.46 (0.42, 0.50)*	0.88 (0.86, 0.90)	0.74 (0.72, 0.77)*	0.59 (0.57, 0.61)	0.60 (0.58, 0.62)
**Female**								
<60			0.37 (0.35, 0.40)	0.38 (0.36, 0.41)	0.84 (0.83, 0.85)	0.84 (0.83, 0.85)	0.60 (0.59, 0.62)	0.61 (0.60, 0.63)*

≥60	0.45 (0.41, 0.48)	0.50 (0.47, 0.54)*	0.76 (0.74, 0.78)	0.70 (0.67, 0.72)*	0.60 (0.58, 0.62)	0.60 (0.58, 0.62)

AUC, area under the ROC curve; CI, confidence interval; CVD, cardiovascular diseases.

*p<0.05 when comparing the traditionally defined metabolic syndrome MetS and the dichotomous age-sex-ethnicity-specific MetS.

Table S10. Characteristics of the participants in the Hubei Behavior and Disease Surveillance (HBDS) survey

Variables	Percentage (%) or Mean±SD					
	Overall (N=20,398)	Male		Female		*p*-value[a]
		**<60 yrs** (N=4,812)	**≥60 yrs** (N=3,159)	**<60 yrs** (N=8,599)	**≥60 yrs** (N=3,828)	
MetS components						
**Waist circumference** (cm)	83.9±9.7	86.8±9.4	85.1±9.9	81.5±9.4	84.4±9.5	<0.001
**Triglycerides** (mmol/L)	1.6±1.0	1.8±1.3	1.4±0.9	1.5±0.9	1.6±0.9	<0.001
**HDL-C** (mmol/L)	1.4±0.4	1.3±0.3	1.4±0.4	1.4±0.3	1.4±0.4	<0.001
**Mean arterial pressure** (mmHg)	99.1±13.0	101.1±12.8	101.4±12.5	96.5±12.8	100.8±12.9	<0.001
**Fasting blood glucose** (mmol/L)	6.0±1.5	6.1±1.6	6.1±1.6	5.9±1.3	6.2±1.7	<0.001
CVD-related risk factors						
**Diabetes**						<0.001
No	18,059 (88.5)	4,228 (87.9)	2,729 (86.4)	7,869 (91.5)	3,233 (84.5)	
Yes	2,339 (11.5)	584 (12.1)	430 (13.6)	730 (8.5)	595 (15.5)	
**Hypertension**						<0.001
No	12,523 (61.4)	3,038 (63.1)	1,514 (47.9)	6,191 (72.0)	1,780 (46.5)	
Yes	7,875 (38.6)	1,774 (36.9)	1,645 (52.1)	2,408 (28.0)	2,048 (53.5)	
**Hyperlipidemia**						<0.001
No	14,601 (71.6)	3,053 (63.4)	2,362 (74.8)	6,494 (75.5)	2,692 (70.3)	
Yes	5,797 (28.4)	1,759 (36.6)	797 (25.2)	2,105 (24.5)	1,136 (29.7)	
CVD-related risk markers						
**Total cholesterol** (mmol/L)	4.8±0.9	4.8±0.9	4.7±0.9	4.8±0.9	5.1±0.9	<0.001
**Body mass index** (kg/m^2)	24.4±3.4	24.8±3.4	23.9±3.2	24.3±3.5	24.5±3.5	<0.001
**Serum uric acid** (μmol/L)	298.9±84.3	346.6±82.5	349.1±87.4	261.8±65.1	287.4±75.0	<0.001
Covariates						
**Marital status**						<0.001
Married	19,107 (93.7)	4,527 (94.1)	2,930 (92.8)	8,367 (97.3)	3,283 (85.8)	
Unmarried/divorced/widowed	1,291 (6.3)	285 (5.9)	229 (7.2)	232 (2.7)	545 (14.2)	
**Educational level**						<0.001
High school or above	5,096 (25.0)	1,737 (36.1)	459 (14.5)	1,784 (20.7)	1,116 (29.1)	
Primary and junior high school	12,045 (59.0)	2,709 (56.3)	1,850 (58.6)	5,011 (58.3)	2,475 (64.7)	

Illiteracy	3,257 (16.0)	366 (7.6)	850 (26.9)	1,804 (21.0)	237 (6.2)	
**Residential location**						0.021
Urban	2,719 (13.3)	609 (12.7)	385 (12.2)	1,211 (14.1)	514 (13.4)	
Rural	17,679 (86.7)	4,203 (87.3)	2,774 (87.8)	7,388 (85.9)	3,314 (86.6)	
**Medical insurance**						0.329
No	351 (1.7)	96 (2.0)	44 (1.4)	146 (1.7)	65 (1.7)	
Yes	20,047 (98.3)	4,716 (98.0)	3,115 (98.6)	8,453 (98.3)	3,763 (98.3)	
**Alcohol drinking**						<0.001
No	14,465 (70.9)	1,905 (39.6)	1,581 (51.0)	7,553 (87.8)	3,426 (89.5)	
Yes	5,933 (29.1)	2,907 (60.4)	1,578 (49.0)	1,046 (12.2)	402 (10.5)	
**Smoking**						<0.001
No	16,547 (81.1)	2,557 (53.1)	1,743 (55.2)	8,524 (99.1)	3,723 (97.3)	
Yes	3,851 (18.9)	2,255 (46.9)	1,416 (44.8)	75 (0.9)	105 (2.7)	
**Insomnia**						<0.001
No	7,876 (38.6)	2,435 (50.6)	998 (31.6)	3,620 (42.1)	823 (21.5)	
Yes	12,522 (61.4)	2,377 (49.4)	2,161 (68.4)	4,979 (57.9)	3,005 (78.5)	
**Diet** (g/week)[b]						
Cereal	2,382.7±1,953.4	2,523.1±2,067.6	2,592.6±2,147.3	2,195.7±1,880.4	2,355.1±2,011.4	<0.001
Fruits	636.3±888.6	603.7±819.4	429.9±672.7	773.0.9±983.9	479.1±761.6	<0.001
Vegetables	3,206.9±1,962.9	3,319.1±1,930.4	3,466.3±2,076.4	3,256.6±1,959.7	3,288.6±2,027.8	<0.001
Meat	538.7±804.6	660.1±860.2	442.5±740.8	486.7±722.3	326.0±663.4	<0.001
**Physical activity**[c]						<0.001
Insufficient	4,426 (21.7)	1,184 (24.6)	900 (28.5)	1,496 (17.4)	846 (22.1)	
Sufficient	15,972 (78.3)	3,628 (75.4)	2,259 (71.5)	7,103 (82.6)	2,982 (77.9)	

CVD, cardiovascular disease; HDL-C, high-density lipoprotein cholesterol; MetS, metabolic syndrome; SD, standard deviation.
[a]Significance of the differences in the values among the four age-sex-specific subgroups, tested by the two-sided analysis of variance for continuous variables and the two-sided Chi-square test for categorical variable. [b]Diet was surveyed as how many grams were consumed on average per week during the preceding 12 months, measured by a mold of the standard serving size. [c]Physical activity was considered sufficient if having at least 150 minutes of moderate aerobic activities or 75 minutes of vigorous aerobic activities per week on average, according to the World Health Organization criteria.

Table S11. Associations between the metabolic syndrome (MetS) score and the cardiovascular disease (CVD)-related risk factors and risk markers in the Hubei Behavior and Disease Surveillance (HBDS) survey

**Groups**	**OR (95% CI)**			**β (95% CI)**		
	**Hyperlipidemia**	**Diabetes**	**Hypertension**	**CHOL**	**BMI**	**SUA**
**Overall**[a]	1.35 (1.34, 1.35)**	1.13 (1.12, 1.14)**	1.20 (1.19, 1.21)**	0.19 (0.17, 0.20)**	2.37 (2.32, 2.41)**	29.34 (27.85, 30.83)**
**Male**[b]						
<60	1.43 (1.41, 1.44)**	1.14 (1.12, 1.15)**	1.20 (1.18, 1.22)**	0.22 (0.18, 0.25)**	2.54 (2.43, 2.64)**	35.48 (31.94, 39.02)**
≥60	1.32 (1.30, 1.34)**	1.12 (1.11, 1.14)**	1.13 (1.10, 1.15)**	0.12 (0.08, 0.16)**	2.16 (2.04, 2.28)**	26.91 (21.39, 32.44)**
**Female**[b]						
<60	1.34 (1.33, 1.35)**	1.10 (1.09, 1.11)**	1.18 (1.16, 1.19)**	0.17 (0.14, 0.20)**	2.06 (1.97, 2.14)**	25.78 (23.74, 27.82)**
≥60	1.31 (1.30, 1.33)**	1.12 (1.10, 1.13)**	1.09 (1.07, 1.11)**	0.06 (0.02, 0.10)*	1.57 (1.44, 1.71)**	25.49 (21.26, 29.72)**

BMI, body mass index; CHOL, cholesterol; CI, confidence interval; HbA1c, glycated hemoglobin; OR, odds ratio; SUA, serum uric acid.

[a]Adjusted for age, sex, marital status, educational level, residential location, medical insurance, alcohol drinking, smoking, insomnia, diet, and physical activity. [b]Adjusted for marital status, educational level, residential location, medical insurance, alcohol drinking, smoking, insomnia, diet, and physical activity.

OR and β are estimated by multiple logistic regression and linear regression, respectively, and all tests are two-sided. $^{*}p<0.05$, $^{**}p<0.001$

Table S12. Associations between the metabolic syndrome (MetS) score in quartiles and the cardiovascular disease (CVD)-related risk factors and risk markers in the Hubei Behavior and Disease Surveillance (HBDS) survey

Groups	Quartiles	OR (95% CI)					
		Hyperlipidemia	Diabetes	Hypertension	Elevated CHOL	Elevated BMI	Elevated SUA
**Overall**[a]	<-0.29	1.00	1.00	1.00	1.00	1.00	1.00
	[-0.29, 0.22)	1.04 (1.03, 1.06)**	1.01 (1.00, 1.03)*	1.11 (1.09, 1.13)**	1.01 (1.00, 1.02)	1.04 (1.03, 1.06)**	1.02 (1.01, 1.04)**
	[0.22, 0.78)	1.23 (1.22, 1.25)**	1.06 (1.05, 1.08)**	1.27 (1.25, 1.29)**	1.03 (1.02, 1.04)**	1.15 (1.14, 1.17)**	1.08 (1.07, 1.10)**
	>0.78	1.92 (1.90, 1.93)**	1.28 (1.27, 1.29)**	1.51 (1.49, 1.53)**	1.08 (1.07, 1.09)**	1.40 (1.39, 1.42)**	1.22 (1.20, 1.23)**
**Male**[b]							
<60	<-0.40	1.00	1.00	1.00	1.00	1.00	1.00
	[-0.40, 0.10)	1.08 (1.05, 1.12)**	1.03 (1.01, 1.06)*	1.09 (1.05, 1.13)**	0.98 (0.96, 1.00)	1.05 (1.02, 1.08)**	1.04 (1.00, 1.07)
	[0.10, 0.62)	1.40 (1.37, 144)**	1.09 (1.06, 1.11)**	1.30 (1.26, 1.34)**	1.01 (0.98, 1.03)	1.20 (1.17, 1.23)**	1.17 (1.13, 1.21)**
	≥0.62	2.18 (2.15, 2.21)**	1.30 (1.27, 1.32)**	1.50 (1.46, 1.54)**	1.08 (1.05, 1.10)**	1.48 (1.45, 1.51)**	1.29 (1.25, 1.33)**
≥60	<-0.43	1.00	1.00	1.00	1.00	1.00	1.00
	[-0.43, 0.14)	1.03 (0.98, 1.07)	1.03 (1.00, 1.07)	1.09 (1.04, 1.15)**	1.00 (0.97, 1.02)	1.02 (0.98, 1.05)	1.01 (0.95, 1.08)
	[0.14, 0.71)	1.18 (1.14, 1.22)**	1.09 (1.06, 1.13)**	1.19 (1.13, 1.25)**	1.01 (0.98, 1.04)	1.05 (1.02, 1.08)*	1.02 (0.96, 1.09)
	≥0.71	1.88 (1.83, 1.92)**	1.06 (1.24, 1.32)**	1.33 (1.27, 1.39)**	1.04 (1.01, 1.07)*	1.31 (1.27, 1.34)**	1.21 (1.15, 1.28)**
**Female**[b]							
<60	<-0.18	1.00	1.00	1.00	1.00	1.00	1.00
	[-0.18, 0.30)	1.03 (1.01, 1.05)*	1.01 (0.99, 1.03)	1.08 (1.05, 1.10)**	1.01 (1.00, 1.03)	1.04 (1.02, 1.06)**	1.02 (1.00, 1.04)
	[0.30, 0.85)	1.15 (1.13, 1.17)**	1.05 (1.03, 1.07)**	1.23 (1.21, 1.26)**	1.02 (1.01, 1.04)*	1.15 (1.13, 1.17)**	1.06 (1.04, 1.08)**
	≥0.85	1.84 (1.82, 1.87)**	1.21 (1.19, 1.23)**	1.45 (1.43, 1.48)**	1.08 (1.06, 1.10)**	1.34 (1.32, 1.36)**	1.15 (1.13, 1.17)**
≥60	<-0.27	1.00	1.00	1.00	1.00	1.00	1.00
	[-0.27, 0.29)	1.03 (1.00, 1.08)	1.01 (0.98, 1.05)	1.09 (1.04, 1.14)**	1.00 (0.97, 1.03)	1.03 (0.99, 1.07)	1.04 (0.99, 1.09)
	[0.29, 0.84)	1.15 (1.11, 1.19)**	1.11 (1.08, 1.15)**	1.09 (1.04, 1.14)**	1.03 (1.00, 1.07)	1.13 (1.08, 1.18)**	1.13 (1.08, 1.18)**
	≥0.84	1.89 (1.85, 1.93)**	1.26 (1.22, 1.29)**	1.22 (1.17, 1.27)**	1.04 (1.01, 1.08)*	1.23 (1.20, 1.27)**	1.21 (1.16, 1.26)**

BMI, body mass index; CHOL, cholesterol; CI, confidence interval; OR: odds ratios; SUA, serum uric acid.

[a]Adjusted for age, sex, marital status, educational level, residential location, medical insurance, alcohol drinking, smoking, insomnia, diet, and physical activity. [b]Adjusted for marital status, educational level, residential location, medical insurance, alcohol drinking, smoking, insomnia, diet, and physical activity.

OR is estimated by multiple logistic regression, and all tests are two-sided. $^{*}p<0.05$, $^{**}p<0.001$

Table S13. Capacities of the traditionally defined metabolic syndrome (MetS) and the dichotomous age-sex-ethnicity-specific MetS in detecting one or more cardiovascular disease (CVD)-related risk factors in the Hubei Behavior and Disease Surveillance (HBDS) survey

**Groups**	**MetS prevalence (95% CI)**		**Sensitivity (95% CI)**		**Specificity (95% CI)**		**AUC (95% CI)**	
	Traditional	Dichotomous	Traditional	Dichotomous	Traditional	Dichotomous	Traditional	Dichotomous
			**Detecting one or more CVD-related risk factors**					
**Overall**	0.37 (0.37, 0.38)	0.41 (0.40, 0.41)*	0.56 (0.55, 0.57)	0.63 (0.62, 0.64)*	0.87 (0.86, 0.87)	0.87 (0.86, 0.88)	0.71 (0.71, 0.72)	0.75 (0.74, 0.75)*
**Male**								
<60	0.41 (0.39, 0.42)	0.40 (0.38, 0.41)	0.60 (0.58, 0.62)	0.61 (0.59, 0.63)	0.88 (0.87, 0.89)	0.92 (0.91, 0.93)*	0.74 (0.73, 0.75)	0.77 (0.76, 0.78)*
≥60	0.31 (0.30, 0.33)	0.38 (0.36, 0.40)*	0.43 (0.41, 0.45)	0.50 (0.48, 0.52)*	0.92 (0.90, 0.94)	0.85 (0.83, 0.87)*	0.68 (0.66, 0.69)	0.68 (0.66, 0.69)
**Female**								
<60	0.36 (0.35, 0.37)	0.34 (0.33, 0.35)*	0.61 (0.59, 0.62)	0.62 (0.61, 0.64)	0.86 (0.85, 0.87)	0.89 (0.88, 0.90)*	0.73 (0.72. 0.74)	0.76 (0.75, 0.77)*
≥60	0.49 (0.47, 0.50)	0.57 (0.56, 0.59)*	0.61 (0.59, 0.62)	0.67 (0.65, 0.69)*	0.77 (0.75, 0.80)	0.64 (0.61, 0.67)*	0.69 (0.67, 0.71)	0.65 (0.64, 0.67)*
			**Detecting one or more CVD-related risk markers**					
**Overall**			0.62 (0.61, 0.63)	0.68 (0.66, 0.69)*	0.73 (0.72, 0.74)	0.70 (0.69, 0.71)*	0.68 (0.67, 0.68)	0.69 (0.68, 0.70)*
**Male**								
<60			0.68 (0.65, 0.70)	0.68 (0.65, 0.70)	0.72 (0.70, 0.74)	0.74 (0.73, 0.76)	0.70 (0.68, 0.71)	0.71 (0.69, 0.73)
≥60			0.50 (0.46, 0.54)	0.59 (0.55, 0.63)*	0.78 (0.76, 0.81)	0.71 (0.69, 0.74)*	0.64 (0.62, 0.67)	0.65 (0.63, 0.67)
**Female**								
<60			0.63 (0.61, 0.66)	0.63 (0.61, 0.65)	0.72 (0.71, 0.74)	0.75 (0.73, 0.76)*	0.68 (0.67, 0.69)	0.69 (0.67, 0.70)
≥60			0.69 (0.66, 0.72)	0.74 (0.71, 0.77)*	0.61 (0.59, 0.63)	0.51 (0.48, 0.53)*	0.65 (0.63, 0.67)	0.62 (0.60, 0.64)*

AUC, area under the ROC curve; CI, confidence interval; CVD, cardiovascular diseases.
*p<0.05 when comparing the traditionally defined metabolic syndrome MetS and the dichotomous age-sex-ethnicity-specific MetS.

Table S14. Characteristics of the participants in the Fujian Behavior and Disease Surveillance (FBDS) survey

Variables	Percentage (%) or Mean±SD					p-value[a]
	Overall (N=40,100)	Male		Female		
		<60 yrs (N=10,997)	≥60 yrs (N=6,509)	<60 yrs (N=15,373)	≥60 yrs (N=7,221)	
MetS components						
**Waist circumference** (cm)	82.2±9.4	84.4±9.2	83.3±9.5	79.9±8.9	82.7±9.4	<0.001
**Triglycerides** (mmol/L)	1.6±1.1	1.8±1.3	1.5±1.0	1.4±1.0	1.6±1.1	<0.001
**HDL-C** (mmol/L)	1.5±0.4	1.4±0.4	1.4±0.4	1.6±0.4	1.5±0.4	<0.001
**Mean arterial pressure** (mmHg)	96.0±12.9	96.8±12.4	99.3±13.2	92.9±12.5	98.2±12.6	<0.001
**Fasting blood glucose** (mmol/L)	5.3±1.4	5.3±1.4	5.5±1.6	5.2±1.3	5.5±1.5	<0.001
CVD-related risk factors						
**Diabetes**						<0.001
No	37,213 (92.8)	10,292 (93.6)	5,859 (90.0)	14,513 (94.4)	6,549 (90.7)	
Yes	2,887 (7.2)	705 (6.4)	650 (10.0)	860 (5.6)	672 (9.3)	
**Hypertension**						<0.001
No	27,997 (69.8)	7,992 (72.7)	3,675 (56.5)	12,195 (79.3)	4,135 (57.3)	
Yes	12,103 (30.2)	3,005 (27.3)	2,834 (43.5)	3,178 (20.7)	3,086 (42.7)	
**Hyperlipidemia**						<0.001
No	26,113 (65.1)	6,590 (59.9)	4,299 (66.0)	11,000 (71.6)	4,224 (58.5)	
Yes	13,987 (34.9)	4,407 (40.1)	2,210 (34.0)	4,373 (28.4)	2,997 (41.5)	
CVD-related risk markers						
**Total cholesterol** (mmol/L)	5.2±1.4	5.1±1.2	5.1±1.3	5.1±1.4	5.5±1.5	<0.001
**Body mass index** (kg/m^2)	23.6±3.4	23.9±3.4	22.9±3.2	23.7±3.4	23.5±3.4	<0.001
Covariates						
**Marital status**						<0.001
Married	36,094 (90.0)	10,249 (93.2)	5,749 (88.3)	14,660 (95.4)	5,436 (75.3)	
Unmarried/divorced/widowed	4,006 (10.0)	748 (6.8)	760 (11.7)	713 (4.6)	1,785 (24.7)	
**Educational level**						<0.001
High school or above	6,791 (16.9)	2,973 (27.0)	666 (10.2)	2,818 (18.3)	334 (4.6)	
Primary and junior high school	18,514 (46.2)	6,464 (58.8)	3,124 (48.0)	7,304 (47.5)	1,622 (22.5)	
Illiteracy	14,795 (36.9)	1,560 (14.2)	2,719 (41.8)	5,251 (34.2)	5,265 (72.9)	

**Residential location**						<0.001
Urban	7,451 (18.6)	2,041 (18.6)	1,034 (15.9)	3,052 (19.9)	1,324 (18.3)	
Rural	32,649 (81.4)	8,956 (81.4)	5,475 (84.1)	12,321 (80.1)	5,897 (81.7)	
**Medical insurance**						<0.001
No	429 (1.0)	124 (1.1)	60 (0.9)	178 (1.2)	67 (0.9)	
Yes	39,671 (99.0)	10,873 (98.9)	6,449 (99.1)	15,195 (98.8)	7,154 (99.1)	
**Alcohol drinking**						<0.001
No	26,873 (67.0)	4,772 (43.4)	3,844 (59.1)	12,133 (78.9)	6,124 (84.8)	
Yes	13,227 (33.0)	6,225 (56.6)	2,665 (40.9)	3,240 (21.1)	1,097 (15.2)	
**Smoking**						<0.001
No	28,737 (71.7)	4,341 (39.5)	2,260 (34.8)	15,126 (98.5)	7,010 (97.0)	
Yes	11,363 (28.3)	6,656 (60.5)	4,249 (65.2)	247 (1.5)	211 (3.0)	
**Insomnia**						<0.001
No	34,496 (86.0)	9,905 (90.1)	5,577 (85.7)	13,394 (87.1)	5,620 (77.8)	
Yes	5,604 (14.0)	1,092 (9.9)	932 (14.3)	1,979 (12.9)	1,601 (22.2)	
**Diet** (g/week)[b]						
Cereal	2,328.3±1447.8	2,519.8±1,537.5	2,565.9±1,520.5	2,142.3±1,361.4	2,202.3±1,341.3	<0.001
Fruits	560.9±1,793.4	527.7±1,760.7	344.3±1,327.0	749.2.6±1,975.6	425.2±1784.4	<0.001
Vegetables	3,009.8±2,902.3	3,015.2±2,990.1	2,945.7±2,884.8	3,090.8±2,876.6	2,895.9±2,831.1	<0.001
Meat	550.3±717.8	711.4±840.6	519.8±683.3	516.2±659.2	405.5±610.2	<0.001
**Physical activity**[c]						<0.001
<4 days/week	25,401 (63.3)	6,850 (62.3)	4,084 (62.7)	9,715 (63.2)	4,752 (65.8)	
≥4 days/week	14,699 (36.7)	4,147 (37.7)	2,425 (37.3)	5,658 (36.8)	2,469 (34.2)	

CVD, cardiovascular disease; HDL-C, high-density lipoprotein cholesterol; MetS, metabolic syndrome; SD, standard deviation.

[a]Significance of the differences in the values among the four age-sex-specific subgroups, tested by the two-sided analysis of variance for continuous variables and the two-sided Chi-square test for categorical variable. [b]Diet was surveyed as how many grams were consumed on average per week during the preceding 12 months, measured by a mold of the standard serving size. [c]Physical activity was measured by asking the question "In your work, farm work, and household activities, how many days per week on average do you have moderate- to high-intensity activities that cause a slight increase in respiration and heart rate?".

Table S15. Associations between the metabolic syndrome (MetS) score and the cardiovascular disease (CVD)-related risk factors and risk markers in the Fujian Behavior and Disease Surveillance (FBDS) survey

Groups	OR (95%CI)			β (95%CI)	
	Hyperlipidemia	Diabetes	Hypertension	CHOL	BMI
**Overall**[a]	1.30 (1.29, 1.31)**	1.06 (1.06, 1.07)**	1.17 (1.16, 1.17)**	0.10 (0.08, 0.11)**	1.93 (1.88, 1.99)**
**Male**[b]					
<60	1.34 (1.33, 1.36)**	1.06 (1.05, 1.06)**	1.16 (1.15, 1.18)**	0.12 (0.10, 0.15)**	2.11 (1.97, 2.25)**
≥60	1.31 (1.29, 1.33)**	1.08 (1.07, 1.09)**	1.18 (1.16, 1.2)**	0.05 (0.01, 0.10)*	1.70 (1.57, 1.83)**
**Female**[b]					
<60	1.29 (1.28, 1.30)**	1.06 (1.05, 1.06)**	1.17 (1.16, 1.18)**	0.13 (0.09, 0.16)**	1.57 (1.50, 1.65)**
≥60	1.25 (1.23, 1.27)**	1.07 (1.07, 1.08)**	1.17 (1.16, 1.19)**	-0.04 (-0.09, 0.00)	1.24 (1.15, 1.33)**

BMI, body mass index; CHOL, cholesterol; CI, confidence interval; HbA1c, glycated hemoglobin; OR, odds ratio; SUA, serum uric acid.

[a]Adjusted for age, sex, marital status, educational level, residential location, medical insurance, alcohol drinking, smoking, insomnia, diet, and physical activity. [b]Adjusted for marital status, educational level, residential location, medical insurance, alcohol drinking, smoking, insomnia, diet, and physical activity.

OR and β are estimated by multiple logistic regression and linear regression, respectively, and all tests are two-sided. $^{*}p<0.05$, $^{**}p<0.001$

Table S16. Associations between the metabolic syndrome (MetS) score in quartiles and the cardiovascular disease (CVD)-related risk factors and risk markers in the Fujian Behavior and Disease Surveillance (FBDS) survey

Groups	Quartiles	OR (95% CI)				
		**Hyperlipidemia**	**Diabetes**	**Hypertension**	**Elevated CHOL**	**Elevated BMI**
**Overall**[a]	<-0.53	1.00	1.00	1.00	1.00	1.00
	[-0.53, -0.02)	1.03 (1.02, 1.05)**	1.01 (1.00, 1.01)*	1.10 (1.09, 1.12)**	1.00 (0.98, 1.01)	1.02 (1.01, 1.03)**
	[-0.02, 0.52)	1.20 (1.19, 1.22)**	1.03 (1.02, 1.03)**	1.23 (1.21, 1.24)**	1.02 (1.01, 1.03)*	1.08 (1.07, 1.09)**
	>0.52	1.80 (1.77, 1.82)**	1.13 (1.12, 1.13)**	1.44 (1.42, 1.45)**	1.06 (1.05, 1.07)**	1.23 (1.22, 1.24)**
**Male**[b]						
<60	<-0.65	1.00	1.00	1.00	1.00	1.00
	[-0.65, -0.18)	1.06 (1.04, 1.09)**	1.00 (0.99, 1.02)	1.10 (1.07, 1.12)**	1.00 (0.98, 1.03)	1.02 (1.00, 1.04)*
	[-0.18, 0.35)	1.32 (1.28, 1.35)**	1.03 (1.01, 1.03)**	1.19 (1.16, 1.22)**	1.01 (0.99, 1.03)	1.08 (1.07, 1.10)**
	≥0.35	2.03 (1.98, 2.08)**	1.10 (1.09, 1.12)**	1.40 (1.36, 1.44)**	1.06 (1.04, 1.09)**	1.29 (1.26, 1.31)**
≥60	<-0.47	1.00	1.00	1.00	1.00	1.00
	[-0.47, 0.06)	1.00 (0.97, 1.03)	1.02 (1.00, 1.03)*	1.09 (1.05, 1.13)**	0.96 (0.93, 0.98)**	1.01 (0.99, 1.03)
	[0.06, 0.60)	1.22 (1.18, 1.26)**	1.06 (1.04, 1.07)**	1.17 (1.12, 1.22)**	1.02 (0.99, 1.05)	1.04 (1.02, 1.06)**
	≥0.60	1.84 (1.78, 1.90)**	1.13 (1.11, 1.13)**	1.28 (1.23, 1.34)**	1.04 (1.01, 1.07)*	1.14 (1.12, 1.17)**
**Female**[b]						
<60	<-0.47	1.00	1.00	1.00	1.00	1.00
	[-0.47, 0.06)	1.02 (0.99, 1.04)	1.00 (0.99, 1.01)	1.08 (1.06, 1.11)**	0.99 (0.97, 1.01)	1.03 (1.02, 1.05)**
	[0.06, 0.60)	1.14 (1.12, 1.17)**	1.02 (1.01, 1.02)*	1.19 (1.16, 1.22)**	1.02 (1.00, 1.04)*	1.10 (1.08, 1.12)**
	≥0.60	1.77 (1.73, 1.81)**	1.10 (1.09, 1.12)**	1.36 (1.33, 1.38)**	1.06 (1.04, 1.08)**	1.24 (1.22, 1.26)**
≥60	<-0.49	1.00	1.00	1.00	1.00	1.00
	[-0.49, 0.07)	0.99 (0.96, 1.03)	1.01 (0.99, 1.03)	1.07 (1.03, 1.11)**	0.97 (0.94, 1.00)	1.03 (1.01, 1.05)*
	[0.07, 0.63)	1.10 (1.06, 1.13)**	1.04 (1.02, 1.05)**	1.11 (1.07, 1.14)**	0.96 (0.93, 0.99)*	1.10 (1.08, 1.12)**
	≥0.63	1.64 (1.59, 1.70)**	1.12 (1.10, 1.13)**	1.20 (1.16, 1.23)**	1.01 (0.98, 1.04)	1.15 (1.12, 1.17)**

BMI, body mass index; CHOL, cholesterol; CI, confidence interval; OR: odds ratios.

[a]Adjusted for age, sex, marital status, educational level, residential location, medical insurance, alcohol drinking, smoking, insomnia, diet, and physical activity. [b]Adjusted for marital status, educational level, residential location, medical insurance, alcohol drinking, smoking, insomnia, diet, and physical activity.

OR is estimated by multiple logistic regression and linear regression, respectively, and all tests are two-sided. $^{*}p<0.05$, $^{**}p<0.001$

Table S17. Capacities of the traditionally defined metabolic syndrome (MetS) and the dichotomous age-sex-ethnicity-specific MetS in detecting one or more cardiovascular disease (CVD)-related risk factors in the Fujian Behavior and Disease Surveillance (FBDS) survey

Groups	MetS prevalence (95% CI)		Sensitivity (95% CI)		Specificity (95% CI)		AUC (95% CI)	
	Traditional	Dichotomous	Traditional	Dichotomous	Traditional	Dichotomous	Traditional	Dichotomous
			**Detecting one or more CVD-related risk factors**					
**Overall**	0.24 (0.24, 0.25)	0.30 (0.29, 0.30)*	0.39 (0.38, 0.39)	0.48 (0.48, 0.49)*	0.93 (0.93, 0.94)	0.93 (0.93, 0.93)*	0.66 (0.66, 0.66)	0.71 (0.70, 0.71)*
**Male**								
<60	0.23 (0.22, 0.24)	0.27 (0.26, 0.28)*	0.39 (0.37, 0.40)	0.47 (0.45, 0.48)*	0.96 (0.95, 0.96)	0.97 (0.96, 0.97)	0.67 (0.66, 0.68)	0.72 (0.71, 0.72)*
≥60	0.23 (0.22, 0.24)	0.33 (0.32, 0.34)*	0.34 (0.32, 0.35)	0.46 (0.44, 0.47)*	0.96 (0.95, 0.96)	0.89 (0.87, 0.90)*	0.65 (0.64, 0.65)	0.67 (0.66, 0.68)*
**Female**								
<60	0.19 (0.19, 0.20)	0.21 (0.21, 0.22)*	0.37 (0.35, 0.38)	0.43 (0.42, 0.45)*	0.94 (0.93, 0.94)	0.95 (0.95, 0.96)	0.65 (0.64, 0.66)	0.69 (0.69, 0.70)*
≥60	0.35 (0.34, 0.37)	0.47 (0.46, 0.48)*	0.45 (0.44, 0.47)	0.56 (0.55, 0.58)*	0.86 (0.84, 0.87)	0.72 (0.71, 0.74)*	0.66 (0.65, 0.66)	0.64 (0.63, 0.65)*
			**Detecting one or more CVD-related risk markers**					
**Overall**			0.41 (0.40, 0.42)	0.47 (0.46, 0.48)*	0.81 (0.81, 0.82)	0.76 (0.76, 0.76)*	0.61 (0.61, 0.62)	0.61 (0.61, 0.62)
**Male**								
<60			0.44 (0.42, 0.46)	0.49 (0.47, 0.51)*	0.83 (0.82, 0.84)	0.79 (0.78, 0.80)*	0.63 (0.62, 0.64)	0.64 (0.63, 0.65)*
≥60			0.40 (0.37, 0.43)	0.49 (0.47, 0.52)*	0.81 (0.80, 0.82)	0.71 (0.70, 0.72)*	0.61 (0.59, 0.62)	0.60 (0.59, 0.62)
**Female**								
<60			0.37 (0.36, 0.39)	0.38 (0.37, 0.40)*	0.86 (0.85, 0.86)	0.83 (0.83, 0.84)*	0.61 (0.61, 0.62)	0.61 (0.60, 0.62)*
≥60			0.46 (0.43, 0.48)	0.53 (0.51, 0.55)*	0.70 (0.68, 0.71)	0.56 (0.55, 0.58)*	0.58 (0.56, 0.59)	0.55 (0.54, 0.56)*

AUC, area under the ROC curve; CI, confidence interval; CVD, cardiovascular diseases.

*$p<0.05$ when comparing the traditionally defined metabolic syndrome MetS and the dichotomous age-sex-ethnicity-specific MetS.

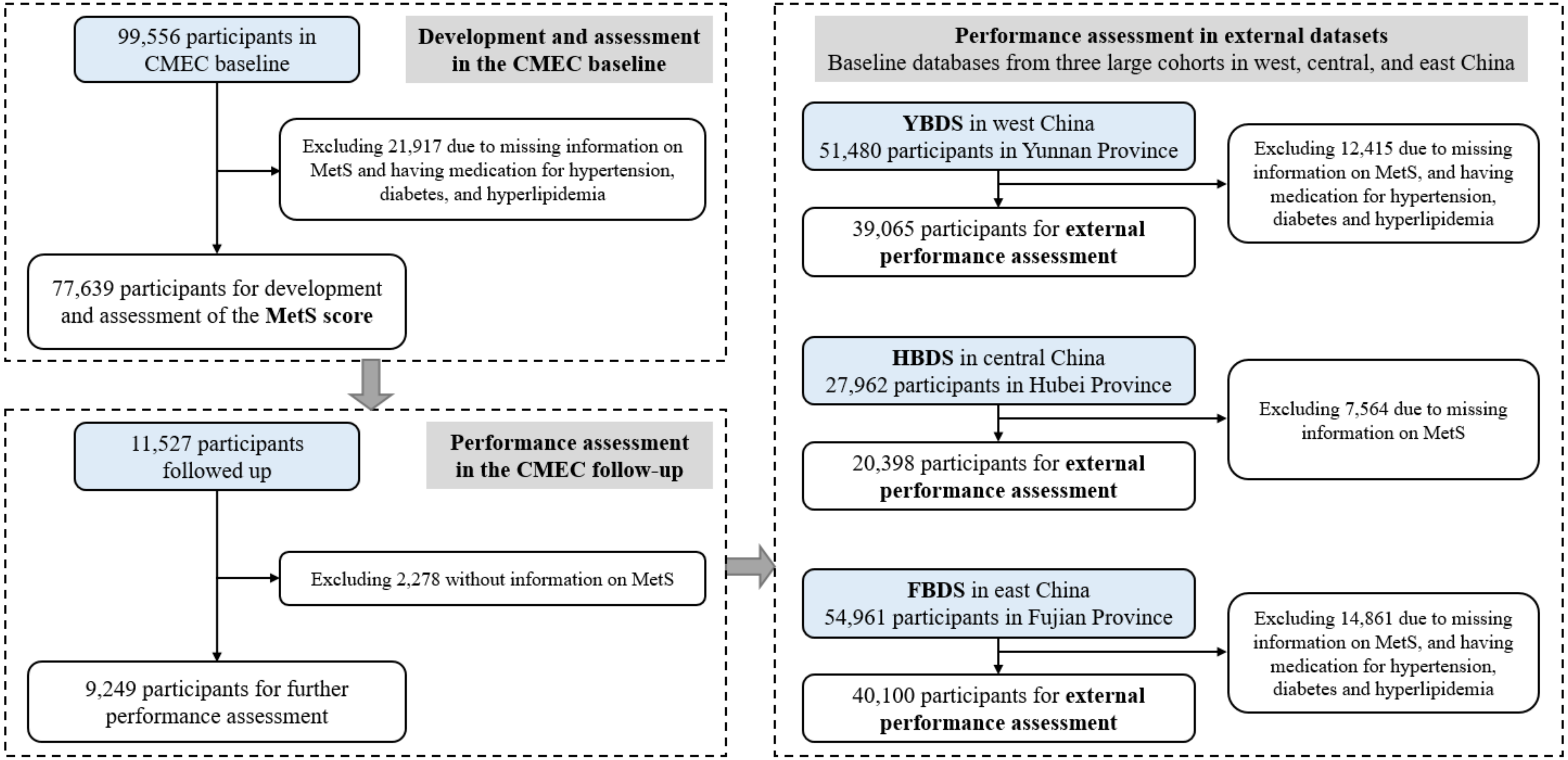

Figure S1. A flowchart of participant enrolment.

CMEC, China Multi-Ethnic Cohort; MetS, Metabolic syndrome; FBDS, Fujian Behavior and Disease Surveillance; HBDS, Hubei Behavior and Disease Surveillance; YBDS, Yunnan Behavior and Disease Surveillance.
