# Supplementary material for: Development and validation of an age-sex-ethnicity-specific metabolic syndrome score in the Chinese adults
Source: Nat Commun. 2023 Nov 1;14:6988. doi: 10.1038/s41467-023-42423-y (PMC10620391; doi:10.1038/s41467-023-42423-y)
Supplement: Supplementary file 3 — Reporting Summary [file 41467_2023_42423_MOESM3_ESM.pdf]

nature portfolio

Corresponding author(s): Peng Jia, Shujuan Yang
Last updated by author(s): Oct 2, 2023

Reporting Summary

Nature Portfolio wishes to improve the reproducibility of the work that we publish. This form provides structure for consistency and transparency in reporting. For further information on Nature Portfolio policies, see our Editorial Policies and the Editorial Policy Checklist.

Statistics

For all statistical analyses, confirm that the following items are present in the figure legend, table legend, main text, or Methods section.

n/a	Confirmed	
[ ]	[x]	The exact sample size (*n*) for each experimental group/condition, given as a discrete number and unit of measurement
[ ]	[x]	A statement on whether measurements were taken from distinct samples or whether the same sample was measured repeatedly
[ ]	[x]	The statistical test(s) used AND whether they are one- or two-sided<br>*Only common tests should be described solely by name; describe more complex techniques in the Methods section.*
[ ]	[x]	A description of all covariates tested
[ ]	[x]	A description of any assumptions or corrections, such as tests of normality and adjustment for multiple comparisons
[ ]	[x]	A full description of the statistical parameters including central tendency (e.g. means) or other basic estimates (e.g. regression coefficient) AND variation (e.g. standard deviation) or associated estimates of uncertainty (e.g. confidence intervals)
[ ]	[x]	For null hypothesis testing, the test statistic (e.g. *F*, *t*, *r*) with confidence intervals, effect sizes, degrees of freedom and *P* value noted<br>*Give P values as exact values whenever suitable.*
[x]	[ ]	For Bayesian analysis, information on the choice of priors and Markov chain Monte Carlo settings
[x]	[ ]	For hierarchical and complex designs, identification of the appropriate level for tests and full reporting of outcomes
[x]	[ ]	Estimates of effect sizes (e.g. Cohen's *d*, Pearson's *r*), indicating how they were calculated

Our web collection on statistics for biologists contains articles on many of the points above.

Software and code

Policy information about availability of computer code

Data collection	No software was used in data collection.
Data analysis	All statistical analyses were completed using R software version 4.0.2 (R Foundation for Statistical Computing).

For manuscripts utilizing custom algorithms or software that are central to the research but not yet described in published literature, software must be made available to editors and reviewers. We strongly encourage code deposition in a community repository (e.g. GitHub). See the Nature Portfolio guidelines for submitting code & software for further information.

Data

Policy information about availability of data

All manuscripts must include a data availability statement. This statement should provide the following information, where applicable:
- Accession codes, unique identifiers, or web links for publicly available datasets
- A description of any restrictions on data availability
- For clinical datasets or third party data, please ensure that the statement adheres to our policy

Data are not accessible to be downloaded publicly because of some sensitive information. Aggregated data may be shared on reasonable request to the corresponding authors.

Research involving human participants, their data, or biological material

Policy information about studies with human participants or human data. See also policy information about sex, gender (identity/presentation), and sexual orientation and race, ethnicity and racism.

Reporting on sex and gender	Findings are sex-specific, which have all been clarified. Sex was determined based on self-reporting. Disaggregated sex data were collected and consent has been obtained, but individual-level data were not shared. Two multi-group one-factor CFA were fit: Model 1 constrained the factor loadings to be equal across the eight combinations of age (young [<60 years] and old [60>=years]), sex (male and female), and ethnicity (Han and minority); Model 2 allowed the factor loadings to vary across the eight subgroups, for developing age-sex-ethnicity-specific MetS scores.
Reporting on race, ethnicity, or other socially relevant groupings	Not applicable.
Population characteristics	Of the 77,639 participants at CMEC baseline, the mean age was 50.2±11.1 years, with 39.0% (30,295) being male and 42.3% (32,857) being minorities. The prevalence of MetS was 19.4% and varied across demographic subgroups, e.g., higher in those aged>=60 than aged <60 (24.2% vs. 18.1%), in males than females (31.0% vs. 17.0%), and in minorities than Hans (19.7% vs. 19.1%).
Recruitment	The CMEC used a multi-stage stratified cluster sampling method to recruit 99,556 participants aged 30-79 years from the five provinces of Southwest China (Sichuan, Chongqing, Yunnan, Guizhou, and Xizang) between May 2018 and September 2019. Among them, about 10% of the subjects in each participating district/county, adding up to 11,527 participants, were selected between August 2020 and July 2021 by a purposive sampling method and followed up in the same way as the baseline survey. A total of 51,480 adults aged >18 years were recruited from 28 districts/counties of Yunnan Province from January to August 2021, by a multi-stage stratified cluster random sampling method.
Ethics oversight	The CMEC was approved by the medical ethic committee of Sichuan University (K2016038). The survey, named as the Yunnan Behavior and Disease Surveillance cohort (YBDS), was approved by the medical ethic committee of Yunnan Center for Disease Prevention and Control (202017).

Note that full information on the approval of the study protocol must also be provided in the manuscript.

Field-specific reporting

Please select the one below that is the best fit for your research. If you are not sure, read the appropriate sections before making your selection.

☐ Life sciences ☒ Behavioural & social sciences ☐ Ecological, evolutionary & environmental sciences

For a reference copy of the document with all sections, see nature.com/documents/nr-reporting-summary-flat.pdf

Behavioural & social sciences study design

All studies must disclose on these points even when the disclosure is negative.

Study description	The CMEC study is an ongoing community-based prospective cohort study, which aimed to examine ethnic variation in the profiles of non-communicable diseases and related risk factors in China. Three provincially representative epidemiological surveys were established in Yunnan, Hubei, and Fujian Province.
Research sample	The CMEC recruited 99,556 participants aged 30-79 years from Sichuan, Chongqing, Yunnan, Guizhou, and Xizang (https://doi.org/10.1093/ije/dyaa185). A total of 99,563 adults aged >18 years were recruited from Yunnan, Hubei, and Fujian Province (https://doi.org/10.1111/all.15706; https://doi.org/10.3389/fpubh.2022.1014380). The sample is representative.
Sampling strategy	A multi-stage stratified cluster sampling method was used. Sample size was determined by existing datasets (https://doi.org/10.1093/ije/dyaa185; https://doi.org/10.1111/all.15706; https://doi.org/10.3389/fpubh.2022.1014380)
Data collection	The surveys of the CMEC, YBDS, HBDS, and FBDS consisted of an electronic questionnaire with face-to-face interviews, medical physical examinations, and clinical laboratory tests. The information on sociodemographics and lifestyles was collected by the electronic questionnaire. The WC was measured 1.0 cm above the navel and to the nearest 0.1 cm with light clothing. The SBP and DBP measurements were performed in a seated, upright position, and were instructed not to smoke, drink alcohol, coffee, or tea, or exercise for at least 30 minutes before the measurement. Both WC and BP were measured three times with a unified instrument, and then the mean values were taken. The venous blood samples were used to measure biochemical blood indexes at least 8 hours after fasting. iPads were used to record data. No one else was present. The researcher was not blinded to the study hypothesis.
Timing	The CMEC used a multi-stage stratified cluster sampling method to recruit 99,556 participants aged 30-79 years from the five provinces of Southwest China (Sichuan, Chongqing, Yunnan, Guizhou, and Xizang) between May 2018 and September 2019. Among them, about 10% of the subjects in each participating district/county, adding up to 11,527 participants, were selected by a purposive sampling method and followed up in the same way as the baseline survey between August 2020 and July 2021.
Data exclusions	A total of 77,639 participants were included, with 21,917 (22.0%) excluded who had missed any MetS component and were taking

antihyperlipidemic, anti-diabetic, or antihypertensive medications. The CMEC follow-up data were used for internal validation of the MetS score, where the 9,249 participants in the follow-up were included after excluding those with incomplete information on the MetS components and diagnosed as CVD-related risk factors at baseline. The YBDS, HBDS, and FBDS data were used for externally independent validation, where the 99,563 participants were included after excluding those with incomplete information on the MetS components, aged <30 years, or taking antihyperlipidemic, anti-diabetic, or antihypertensive medications.

Non-participation

The response rates of CMEC, YBDS, HBDS, and FBDS are about 60%, 85%-92%, 91%, and 99.7%, respectively.

Randomization

Randomization was not applicable to the study as this is an observational study.

Reporting for specific materials, systems and methods

We require information from authors about some types of materials, experimental systems and methods used in many studies. Here, indicate whether each material, system or method listed is relevant to your study. If you are not sure if a list item applies to your research, read the appropriate section before selecting a response.

Materials & experimental systems

n/a	Involved in the study	
☒	☐	Antibodies
☒	☐	Eukaryotic cell lines
☒	☐	Palaeontology and archaeology
☒	☐	Animals and other organisms
☒	☐	Clinical data
☒	☐	Dual use research of concern
☒	☐	Plants

Methods

n/a	Involved in the study	
☒	☐	ChIP-seq
☒	☐	Flow cytometry
☒	☐	MRI-based neuroimaging
